# A rationally designed bicyclic peptide remodels Aβ42 aggregation in vitro and reduces its toxicity in a worm model of Alzheimer’s disease

**DOI:** 10.1038/s41598-020-69626-3

**Published:** 2020-09-17

**Authors:** Tatsuya Ikenoue, Francesco A. Aprile, Pietro Sormanni, Francesco S. Ruggeri, Michele Perni, Gabriella T. Heller, Christian P. Haas, Christoph Middel, Ryan Limbocker, Benedetta Mannini, Thomas C. T. Michaels, Tuomas P. J. Knowles, Christopher M. Dobson, Michele Vendruscolo

**Affiliations:** 1 Centre for Misfolding Diseases, Department of Chemistry, University of Cambridge, Cambridge, CB2 1EW UK; 2 Present Address: Department of Chemistry, The University of Tokyo, 7-3-1 Hongo, Bunkyo-ku, Tokyo 113-0033 Japan; 3 Present Address: Department of Chemistry, Molecular Sciences Research Hub, Imperial College London, London, W12 0BZ UK; 4 Present Address: Department of Chemistry and Life Science, United States Military Academy, West Point, NY, 10996 USA

**Keywords:** Chemical biology, Drug discovery

## Abstract

Bicyclic peptides have great therapeutic potential since they can bridge the gap between small molecules and antibodies by combining a low molecular weight of about 2 kDa with an antibody-like binding specificity. Here we apply a recently developed in silico rational design strategy to produce a bicyclic peptide to target the C-terminal region (residues 31–42) of the 42-residue form of the amyloid β peptide (Aβ42), a protein fragment whose aggregation into amyloid plaques is linked with Alzheimer’s disease. We show that this bicyclic peptide is able to remodel the aggregation process of Aβ42 in vitro and to reduce its associated toxicity in vivo in a *C. elegans* worm model expressing Aβ42. These results provide an initial example of a computational approach to design bicyclic peptides to target specific epitopes on disordered proteins.

## Introduction

The amyloid β peptide (Aβ) is an intrinsically disordered protein fragment that readily self-assembles into amyloid fibrils, which are the major components of the amyloid plaques that represent a molecular hallmark of Alzheimer’s disease^1–5^. While inhibiting the aggregation of Aβ has been pursued as a major therapeutic strategy against Alzheimer’s disease^2–10^, molecules with clinical efficacy have not yet become available^11–13^, thus prompting the search for novel types of compounds with potential clinical efficacy.

Quite generally, low molecular weight compounds have the advantages of low manufacturing costs and high cell membrane permeability, which enable intracellular targeting^14–16^, and the disadvantages of typically low specificity, high risk of side effects, and a lower ability to inhibit protein–protein interactions. On the other hand, large biomolecules such as antibodies and other biologics have the advantage of high specificity, while they have the disadvantages of high manufacturing costs, difficulty for administration, low permeability and sometimes poor developability^17,18^.

**Figure 1:**
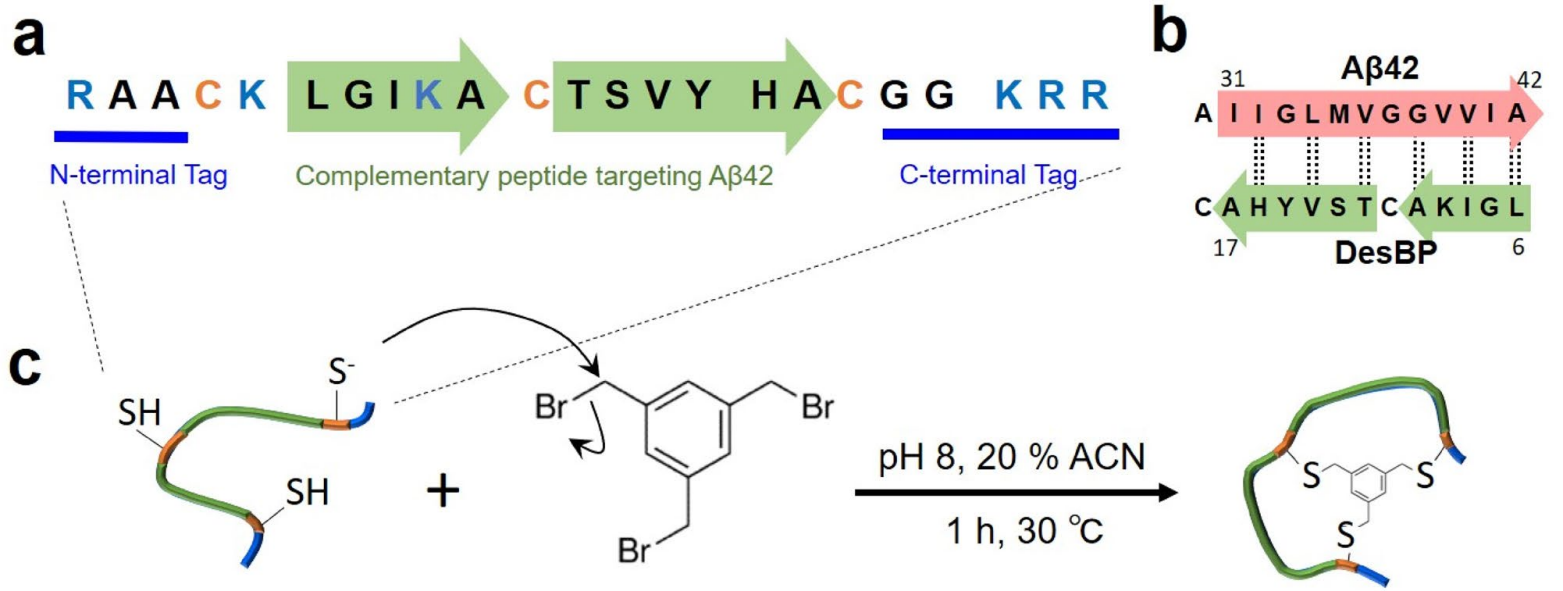
Sequence and synthesis of DesBP, the rationally designed bicyclic peptide described in this work. (**a**) A 23-residue sequence was rationally designed to bind the C-terminal region (residues 31–42) of Aβ42 through two binding regions (green arrows); three cysteine residues were inserted for cyclization (orange) and six positively charged residues (blue) were added outside the binding regions to improve the solubility of the designed sequence for the bicyclic peptide (DesBP). (**b**) Representation of the designed binding mode of DesBP. Dotted lines mark residues predicted to be involved in backbone-backbone hydrogen bonding and arrows denote the N- to C-terminus direction. (**c**) Synthesis of DesBP. The rationally-designed 23-residue peptide was tethered through its three cysteine residues to the trifunctional compound 1,3,5-tris(bromomethyl)benzene (TBMB) in a nucleophilic substitution reaction (see “Materials and methods” section).

To combine the advantages of small molecules with those of antibodies, bicyclic peptides have recently been introduced in the drug discovery field^14,19–24^. Bicyclic peptides are polypeptide chains in which three cysteine residues spaced within the sequence are chemically linked to a cyclic compound, resulting in the formation of two macrocyclic rings, which can act as binding regions (Fig. 1). The structure of a bicyclic peptide is conformationally restrained, leading to a relatively small entropy cost upon binding and thus to a good binding affinity and specificity^23–25^. The small size of bicyclic peptides (about 2 kDa) provides, at least in principle, multiple advantages over antibodies, including the possibility of simple chemical synthesis, better tissue penetration, higher resistance to protease cleavage and inactivation, and extended half-life in vivo^26^. Evidence is also emerging that bicyclic peptides can be developed to be able to cross the blood–brain barrier^27,28^.

Various discovery strategies are currently available for the discovery of bicyclic peptides against given targets. Phage display, in particular, is often used for the isolation of antibodies and bicyclic peptides from large combinatorial libraries^24,29,30^. In some cases, however, this method can be time-consuming and ineffective, particularly if one is interested in targeting weakly immunogenic epitopes or aggregation-prone antigens. To overcome these limitations in the case of antibodies, we previously introduced a method to rationally design antibodies targeting specific epitopes within intrinsically disordered proteins^31–34^.

In this study, we apply this design strategy to generate a bicyclic peptide capable of binding Aβ42 and of interfering with its aggregation process. The aggregation of Aβ42 is a complex process resulting from the combination of different microscopic steps and involving a variety of molecular species^35,36^. In particular, increasing evidence suggests that Aβ42 oligomers, which are formed during the aggregation process, are highly cytotoxic^37,38^. Therefore, some therapeutic strategies aim at decreasing the concentrations of these oligomeric species by delaying or preventing their formation^32,35,37,39–44^. In addition, strategies based on reducing the concentration of toxic oligomers by enhancing the rate of aggregation have also been proposed^45–47^. In particular, several attempts have also focused on redirecting the amyloid aggregation towards off-pathway species of lower toxicity, which could be in principle more safely removed by clearance mechanisms such as microglia-mediated phagocytosis or autophagy. The small molecules epigallocatechin gallate (EGCG)^48,49^ and trodusquemine^50^, for example, can modulate the aggregation process of a range of amyloidogenic peptides and proteins, including islet amyloid polypeptide, Aβ and α-synuclein, by redirecting them towards the formation of non-toxic aggregates^48,49,51,52^.

In this context, we show here that our rationally designed bicyclic peptide affects the formation of toxic species of Aβ42 both in vitro and in vivo using a *C. elegans* model of Aβ42-mediated toxicity by redirecting the aggregation pathway of Aβ42 towards the formation of non-toxic species.

## Results

### Rational design and synthesis of DesBP, a bicyclic peptide targeting Aβ42

As the available structures of Aβ42 amyloid fibrils indicate that the C-terminus of this peptide is involved in the cross-β core of these structures^53–55^, we employed a recently developed rational design strategy to obtain a bicyclic peptide targeting this region (see “Materials and methods” section). A series of complementary peptides were designed to bind this target region using the cascade method^31^, a fragment-based procedure that exploits amino acid sequence fragments known to interact within experimentally-derived protein structures.

Designed peptide candidates were then screened in silico for solubility using the CamSol method^56^, and one sequence (Fig. 1a, b) offering a good compromise between solubility and complementarity scores^41^ was selected for synthesis. The screening for solubility is particularly important for bicyclic peptides, as the requirement of having three cysteine residues within the short peptide sequence (Fig. 1c) and the hydrophobic nature of the scaffold often pose strong limitations on the solubility of these peptides. At variance with other methods of computational design, the approach that we employed here does not require any structural information, but only the knowledge of the amino acid sequence of the target. Furthermore, the success rate of this design strategy is very high, as all the designed antibodies experimentally tested so far showed binding towards their targets^31–34^.

The resulting designed linear peptide was prepared by solid-phase synthesis (see “Materials and methods” section). Then, since the cyclisation achieved via a reducible disulfide bond may not be suitable for therapeutic uses, we used the small organic compound tris(bromomethyl)benzene (TBMB) as a scaffold to anchor the designed peptide containing three cysteine residues (Fig. 1c)^29,57,58^. The reaction occurs in aqueous solvents at 30 °C in 1 h, and the threefold rotational symmetry of the TBMB molecule ensures the formation of a unique structural and spatial isomer. The synthesized bicyclic peptide (DesBP) showed high purity (> 95%). Static light scattering measurements were performed (Supplementary Fig. 1), suggesting that this compound is in a monomeric state in phosphate buffer.

### Characterisation of the interaction of DesBP with Aβ42 monomers

As a first step, we tested whether or not DesBP was able to interact with Aβ42 monomers. Aβ42 is disordered in its monomeric form, as it does not readily adopt one single, stable conformation as a result of its highly dynamical nature. While it is increasingly reported that some small compounds can inhibit their aggregation of this peptide, it is still unclear whether they do so by interacting with the monomeric form^59,60^.

**Figure 2:**
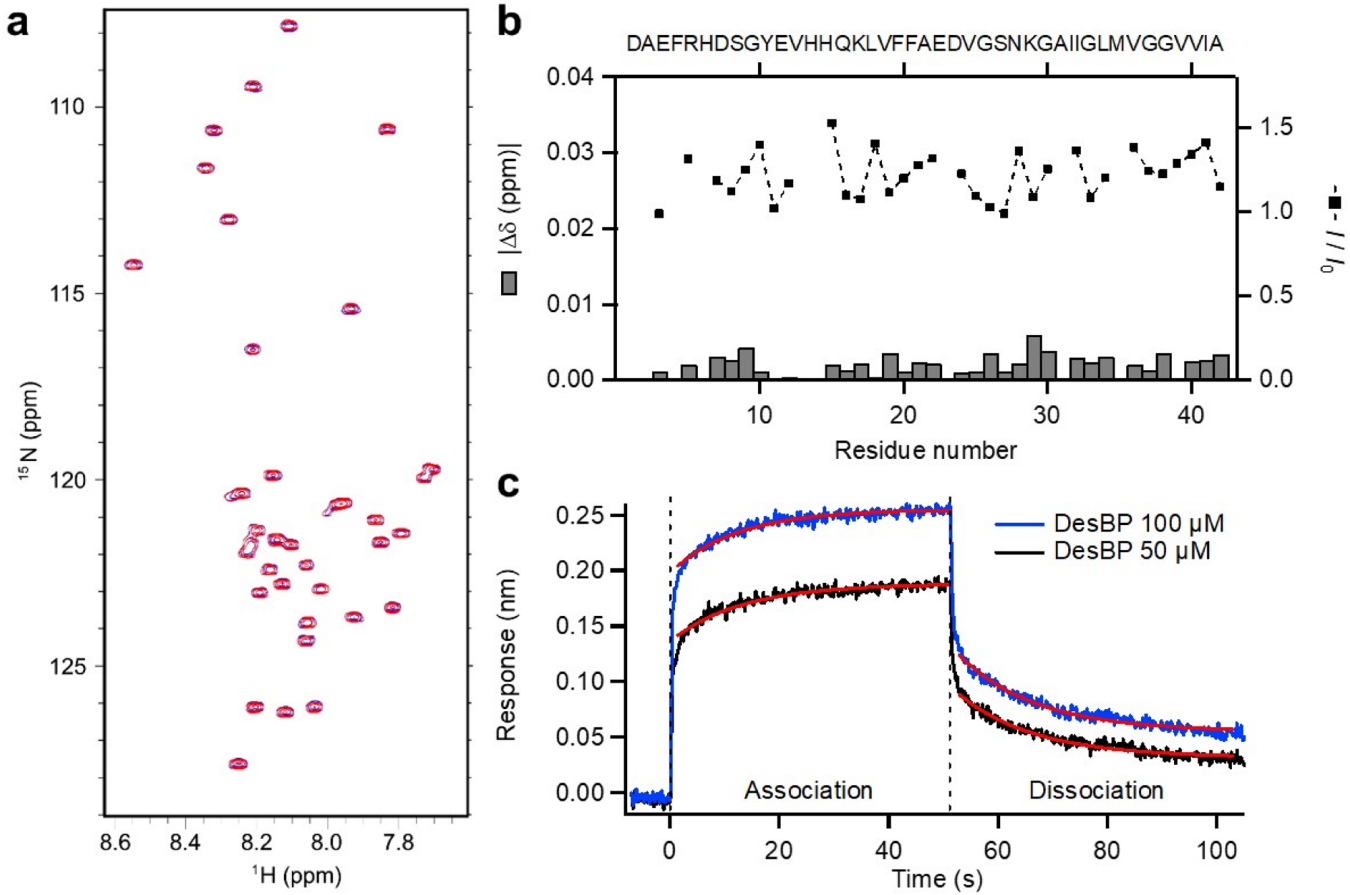
DesBP weakly interacts with monomeric Aβ42. (**a**) ^1^H-^15^N-HSQC spectrum of 15 μM ^15^N-labeled Aβ42 monomers in the absence (blue) and presence (red) of 240 μM DesBP. 32 scans were taken for each spectra at 5 °C on a 500 MHz NMR. (**b**) Chemical shift differences (bar graph) and normalised intensity (dotted line) of Aβ42 in the presence of DesBP suggest minimal interaction of DesBP with monomeric Aβ42. (**c**) BLI binding assay showing the dynamic association and dissociation processes between the Aβ42 and DesBP at the concentrations of 50 μM (black) and 100 μM (blue). The dashed line represents the time at which the BLI sensor was transferred to the control buffer. The kinetic profile of association and dissociation were well fitted by single-exponential functions (red line). The dissociation constant was estimated to be 640 ± 260 μM (association rate k_a_ = 0.102 ± 0.003 M^-1^ s^−1^, dissociation rate k_d_ = 0.060 ± 0.002 s^−1^).

To characterise the binding of DesBP to the monomeric state of Aβ42, we used nuclear magnetic resonance (NMR) spectroscopy, isothermal titration calorimetry (ITC), and bio-layer interferometry (BLI). By performing ^1^H-^15^N HSQC experiments at 5 °C with 15 μM of ^15^N-labeled Aβ42 in presence and absence of 240 μM DesBP (Fig. 2a), we found that the presence of DesBP did not create major differences in the HSQC spectra (Fig. 2b), implying that the interaction between Aβ42 and DesBP is transient and weak, as it is often the case of small compounds with disordered proteins^59–62^.

Next, we performed ITC measurements in which 1 mM of DesBP solution in a syringe was titrated to 10 μM of Aβ42 monomer solution in sample cell at 15 °C (Supplementary Fig. 2). Our results showed very low Δ*H* values, indicating a weak interaction between DesBP and Aβ42, which is consistent with the results of the HSQC experiments. Circular dichroism (CD) spectrometry showed that Aβ42 remained in its monomeric state after the ITC measurements. We then used BLI to measure the kinetics of binding of DesBP and Aβ42. Aβ42 monomers biotinylated at the N-terminus were anchored to the BLI sensor tip and addition of 50 and 100 μM of DesBP rapidly increased the optical interference signals with concentration dependence (Fig. 2c). The global fitting of the kinetic profiles suggested a dissociation constant (*K*_D_) value of 640 $\pm \hspace{0.17em}$260 μM.

Taken together, these results suggest that DesBP interacts, albeit weakly and transiently, with the monomeric form of Aβ42. This observation is in agreement with previous finding in which small molecules^59,60^ and antibodies^31,32^ interact weakly with Aβ42 monomers, but can still strongly inhibit Aβ42 aggregation.

### Characterisation of the effects of DesBP on the aggregation process of Aβ42

In order to investigate the effects of DesBP on the aggregation of Aβ42, we carried out in vitro aggregation assays using thioflavin T (ThT) as an amyloid-specific fluorescent probe^63,64^. We monitored fibril formation for Aβ42 at a concentration of 2 µM in the absence and presence of a range of concentrations (0.25–16 fold excess) of DesBP at 37 °C under quiescent conditions, using a highly reproducible protocol previously described^65^. We then validated our results by means of far-UV circular dichroism (CD) and atomic force microscopy (AFM).

**Figure 3:**
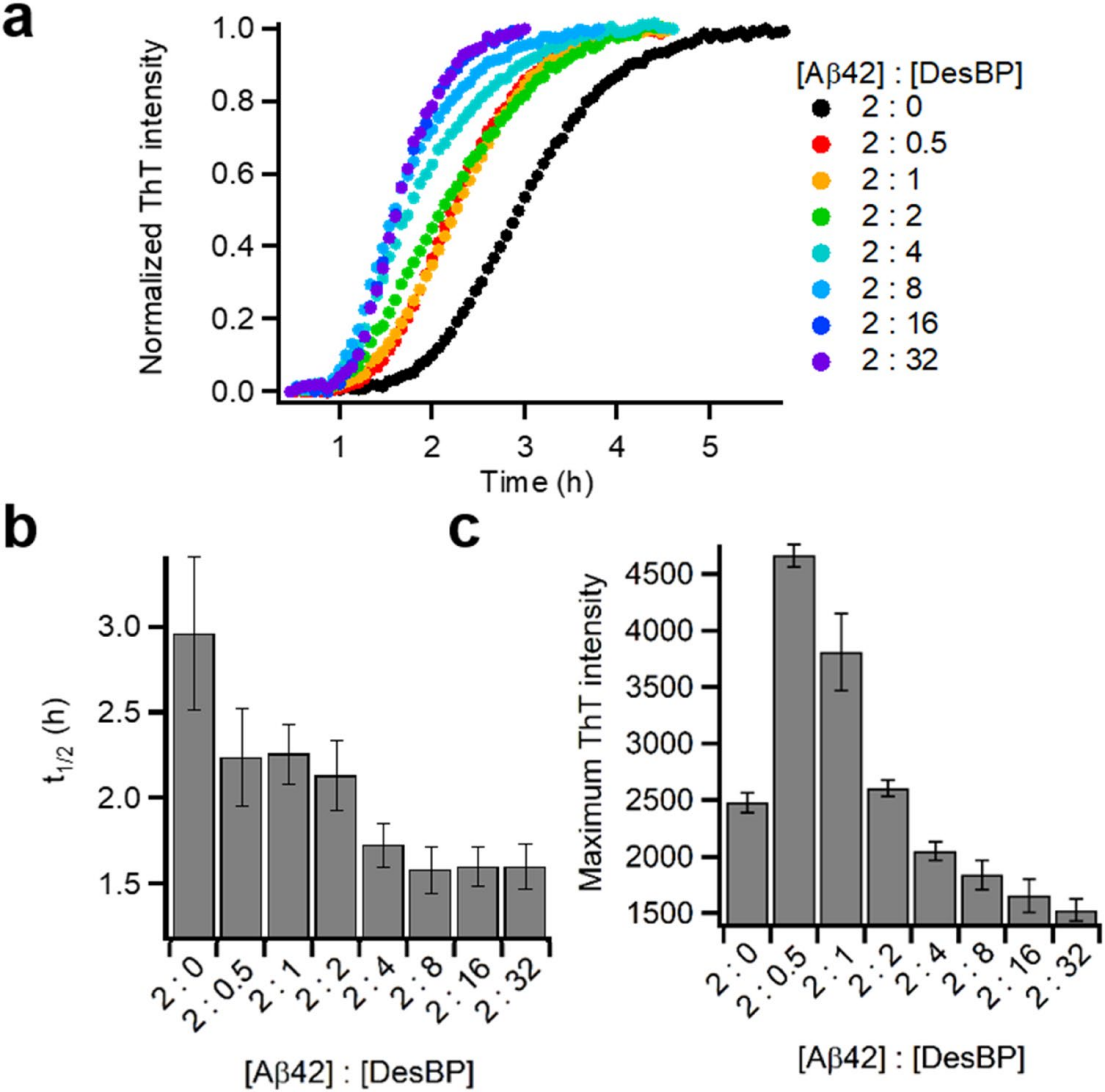
DesBP modifies the aggregation process of Aβ42. (**a**) Normalised kinetic profiles of Aβ42 aggregation under quiescent conditions at a concentration of 2 μM in the absence and presence of various concentrations of DesBP, represented by different colors. (**b**) Average half-time of the aggregation at decreasing [Aβ42]:[DesBP] ratios. (**c**) Average maximum ThT fluorescence intensity of the aggregation at decreasing [Aβ42]:[DesBP] ratios. In (**b**, **c**), the error bars represent the standard deviation over 5 replicates.

We observed that the aggregation rate changed as a function of the concentration of DesBP, as the addition of increasing concentrations of DesBP accelerated the aggregation of Aβ42 (Fig. 3 and Supplementary Fig. 3a). In order to obtain more quantitative information on the effects of DesBP on Aβ42 aggregation, we normalised each aggregation curve (Fig. 3a) for the respective maximum fluorescence value and we derived the half-time of aggregation (*t*_1/2_) and the lag time (Fig. 3b). We found that DesBP leads to a systematic reduction of *t*_1/2_ with increasing DesBP concentrations. Furthermore, small quantities (0.25 molar equivalents) of DesBP were able to produce a reduction of about 25% of the lag time (from 2.9 to 2.2 h) in the aggregation of Aβ42 (Fig. 3b).

Next, we applied a kinetic analysis to obtain more insight into the microscopic steps of aggregation most affected by DesBP. We thus evaluated the changes in the parameters *k*_+_*k*_*n*_ and *k*_+_*k*_2_ of Aβ42 aggregation in the presence of DesBP, where *k*_+_, *k*_*n*_, and *k*_2_ are the rate constants for elongation, primary, and secondary nucleation, respectively^7^. These parameters in the presence of DesBP were determined by fitting the normalised spontaneous aggregation curves (Supplementary Fig. 3b). The results that we obtained using this kinetic model indicate that both primary and secondary nucleation are accelerated (*k*_+_*k*_*n*_ is increased by a factor 2.1 and *k*_+_*k*_2_ by a factor 1.6) in the presence of 0.25 molar equivalents of DesBP, while the elongation rate is not affected significantly by DesBP (Supplementary Fig. 3b).

The highest value of the ThT fluorescence was also affected by the presence of the DesBP (Fig. 3c), while DesBP itself did not affect the ThT signal. In the presence of concentrations of DesBP below 2 molar equivalents, the ThT fluorescence reached values higher than in the absence of DesBP and it became lower as the concentration was increased (Fig. 3c). These results did not depend significantly on the concentration of Aβ42 (Supplementary Fig. 4a–c). The magnitude of the ThT fluorescence at increasing Aβ42:DesBP ratios (2:1, 1:0, 1:2) was linearly correlated with concentration of Aβ42 (Supplementary Fig. 4d).

**Figure 4:**
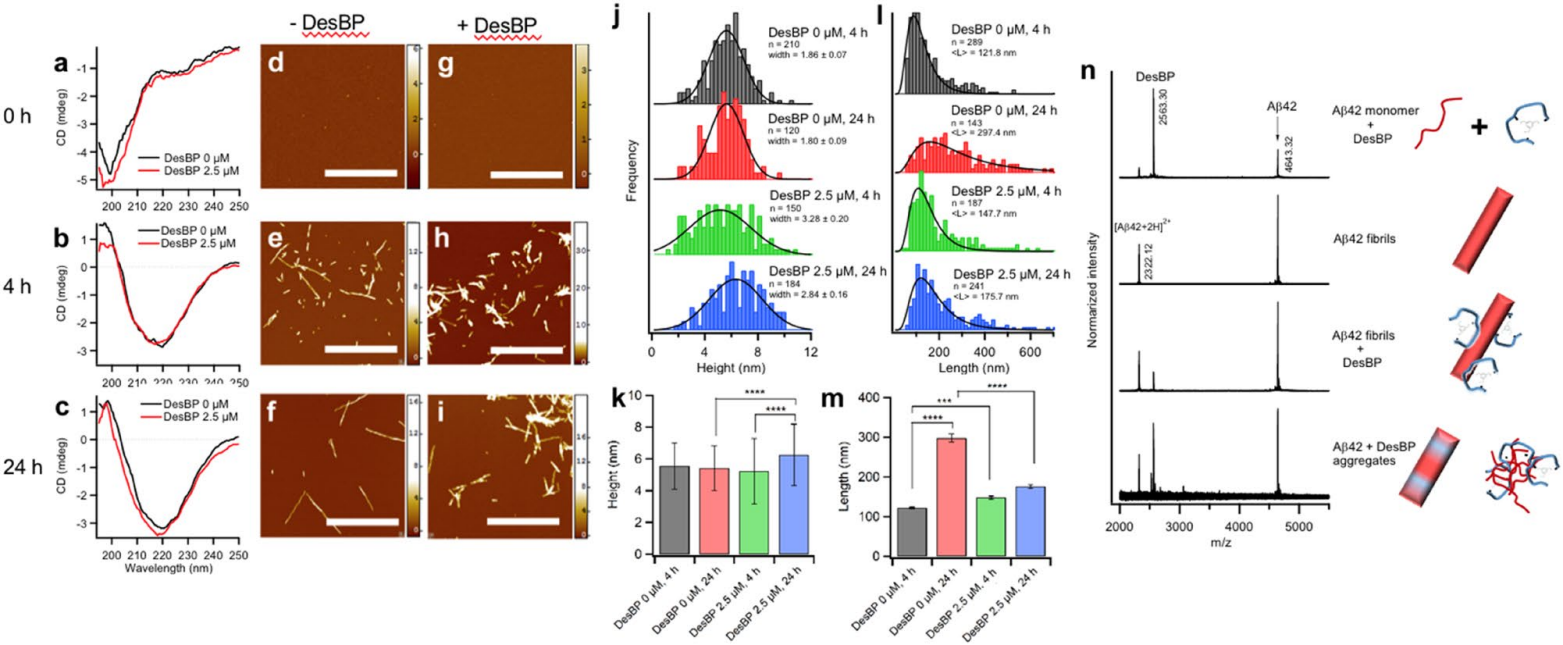
Structural features and population of Aβ42 aggregates at low concentration of DesBP. (**a**–**c**) Far-UV CD spectrum of 10 μM Aβ42 aggregates in absence (black) and presence (red) of 2.5 μM DesBP at three time points 0 (**a**), 4 (**b**), and 24 h (**c**). (**d**–**i**) Representative AFM images of Aβ42 in the absence of DesBP at different incubation times [0 (**d**), 4 (**e**) and 24 h (**f**)], and in the presence of 2.5 μM of DesBP at corresponding incubation times [0 (**g**), 4 (**h**) and 24 h (**i**)]. The scale bar on the AFM images indicate 1 μm and scale exhibited at the right represents the height. (**j**–**m**) Distribution of thickness (**j**) and length (**l**) and the mean values of height (**k**) and length (**m**) of fibrils at different time points (0, 4, and 24 h) formed in absence and presence of 2.5 μM determined by statistical analysis of AFM images. The n values in the histogram represent the number of fibrils used for AFM analysis. The symbols *, **, and ****p < 0.05, 0.01, 0.001, respectively. Fitted Gaussian curves are drawn as eye-guide in (**j**). Curves in (**l**) are fits with the log-normal distribution function (Eq. 1) and calculated mean values are represented with red bars in (**m**). Error bars of arithmetic averages of height (**k**) and length [black bars in (**m)**] represent STD and SEM, respectively. (**n**) MALDI mass spectrometry of aggregated contents. Samples were prepared at concentration of 5 μM Aβ42 with or without 20 μM DesBP. Aggregates are dissociated by 8 M Gdn-HCl at pH 8.0.

After reaching maximum intensity, the ThT fluorescence gradually decreased with the time of incubation (Supplementary Fig. 3a). To explore whether DesBP inhibits ThT binding or promotes Aβ42 amorphous fibril precipitation, resulting in a decrease of the ThT fluorescence, we performed a kinetic assay in which DesBP and ThT were added after the formation of Aβ42 fibrils (Supplementary Fig. 5). The kinetic profile did not show significant differences between the presence and absence of DesBP, indicating that DesBP does not affect the already formed amyloid fibrils, and suggesting that aggregates formed in the presence of DesBP might have different properties that induce the continuous decrease of the ThT fluorescence. We further analysed the decrease in the ThT signal (Supplementary Fig. 3) using normalised ThT profiles (Supplementary Fig. 6). As DesBP concentration increased, the ThT intensity decreased, suggesting that fibrils formed in the presence of DesBP are increasingly prone to self-association and to form larger precipitates.

**Figure 5:**
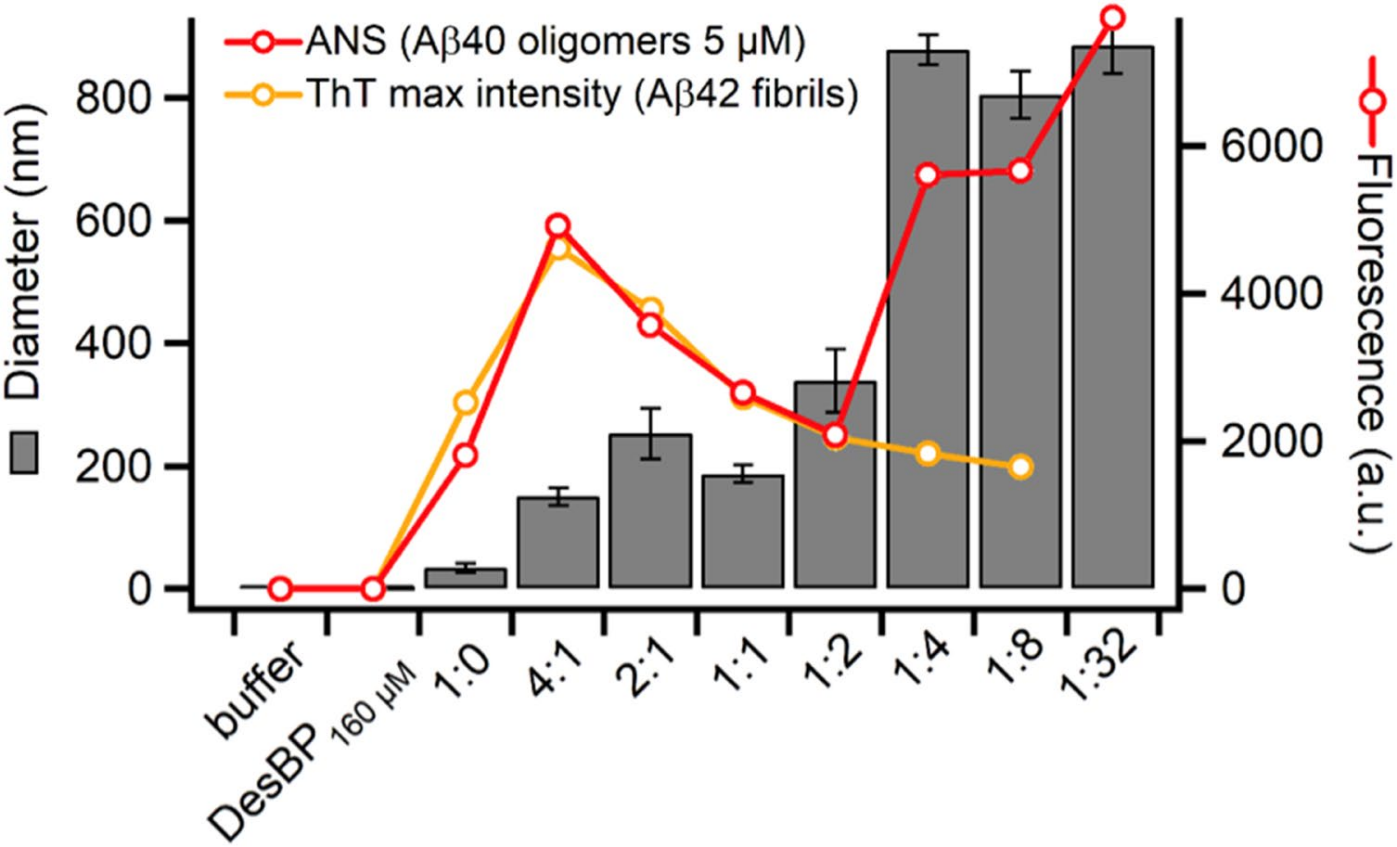
Effects of DesBP on the morphology of  Zn^2+^-stabilised oligomers [38]. ANS fluorescence (red line) and size (grey bars) of Aβ40 oligomers at a concentration of 5 μM in the absence or in the presence of various concentrations of DesBP. Zn^2+^-stabilised oligomers were incubated for 2 h after addition of DesBP at room temperature. The maximum of the ThT fluorescence from the kinetic measurements is shown for comparison (orange).

**Figure 6:**
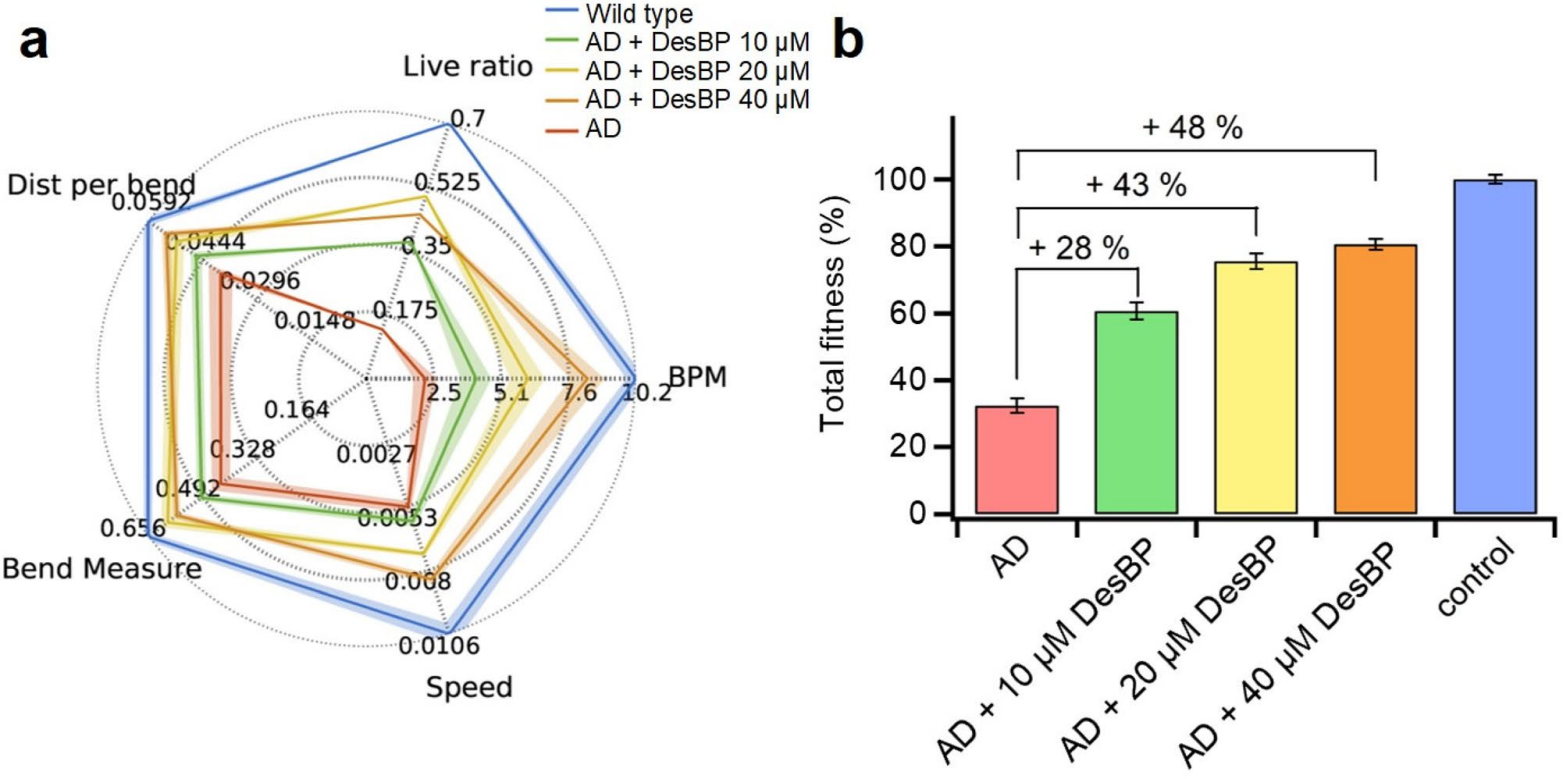
DesBP restores the motility in a *C. elegans* model of Aβ42-mediated toxicity. (**a**) Fingerprint of the measurements of the effect of increasing concentration of DesBP, from 0 μM (red), to 10 μM (green), 20 μM (yellow) and 40 μM (orange) on the motility of the GMC1010 worm model of Alzheimer’s disease used in this work; N2 worms treated with lipid vesicles (blue) and with DesBP (Fig. S12, green) were used as controls. The fitness of the worms was measured by 5 different readouts: the fraction of worms alive at the end of the experiments (live ratio), the average number of body bends per minute (BPM), the average speed of movement (speed), the average amplitude of the bend motion (Bend Measure), and the average distance traveled per bend (Dist per bend). (**b**) The five fitness parameters in panel a were combined in an overall fitness parameter (total fitness) of the worms^78^ in the presence and absence of DesBP.

To reveal the effects of the TBMB scaffold of DesBP on the aggregation of Aβ42, we performed ThT assays with the designed linear peptide in its non-cyclic form and with 1,3,5-trimethylbenzene (TMB), which mimics the linker part (TBMB) in DesBP. The linear peptide showed similar effects on the Aβ42 aggregation (Supplementary Fig. 7a), while TMB slightly delayed the nucleation step of Aβ42 aggregation and did not show significant changes on the magnitude of ThT fluorescence (Supplementary Fig. 7b) indicating a different inhibition mechanism. These results support the conclusion that the effects of DesBP that we observed on Aβ42 aggregation depend more strongly on the designed peptide component than on TBMB.

**Figure 7:**
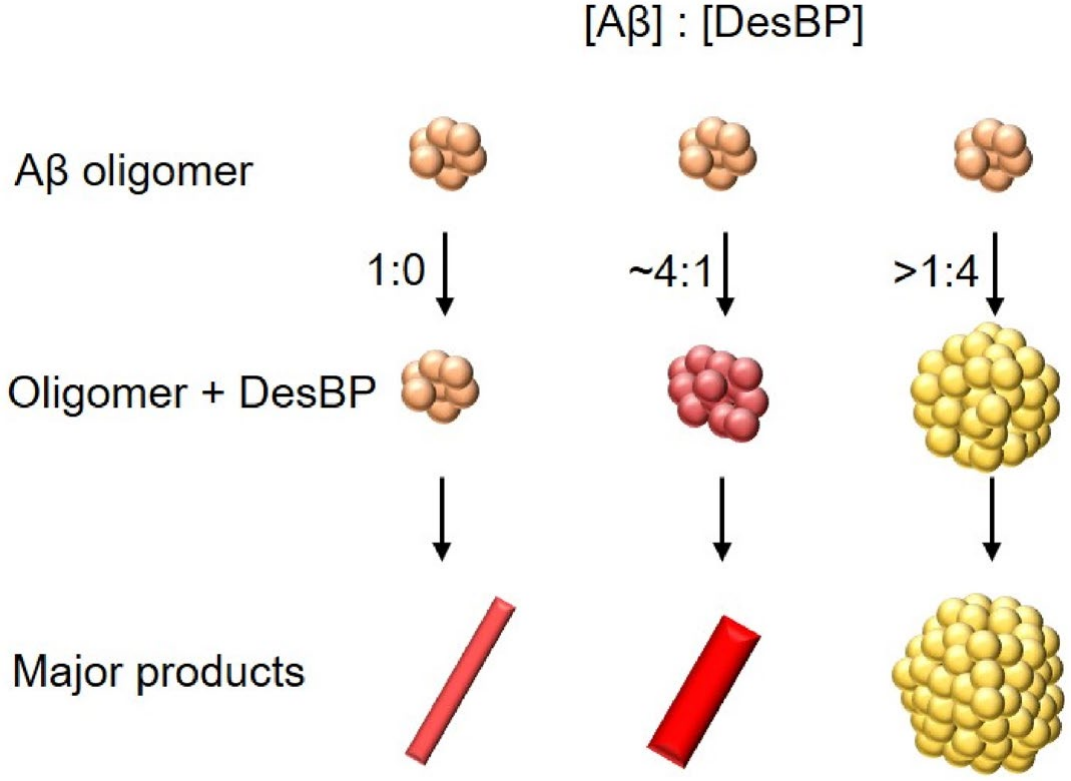
Effects of DesBP on the aggregation process of Aβ42. Depending on the Aβ42:DesBP ratio, our results indicate increasing effects of DesBP on the morphology of Aβ42 aggregates, from DesBP containing fibrillar assemblies (Fig. 4) to more disordered deposits (Figure S9).

Taken together, these results show that DesBP interferes with Aβ42 aggregation by promoting the formation of ThT-sensitive early species and by inhibiting the formation of ThT-sensitive late species.

### Characterisation of the effects of DesBP on the morphology of Aβ42 aggregates

At low DesBP concentrations (for Aβ42:DesBP ratios above 1:1) the ThT intensity was increased at the early stages of the aggregation process (Supplementary Fig. 3a), although the CD spectra at different time points during the aggregation process did not show significant differences between the samples in the presence and absence of DesBP (Fig. 4a–c). Since the amount of soluble Aβ42 monomers after the formation of amyloid fibrils (i.e. the solubility of Aβ42) was not changed by the presence of DesBP (Supplementary Fig. 8), at least at Aβ42:DesBP ratios above 4:1, the gain of ThT intensity is not likely to represent the changes of the aggregate mass, but it may rather be caused by some structural changes of the aggregates themselves. To investigate this possibility, supernatants of 2 μM of Aβ42 at several time points in the presence of 0, 0.25, and 2 molar equivalents of DesBP were analysed by reversed phase chromatography (Supplementary Fig. 8). The total peak area of Aβ42 showed that 2 molar equivalents of DesBP increased the residual monomer concentration of Aβ42, implying that the aggregate mass is reduced or that the aggregates become thermodynamically less stable, while 0.25 molar equivalents of DesBP did not make significant changes. Since the aggregate mass at 24 h was not significantly changed (Supplementary Fig. 8b) from 5 h, the continuous decrease of the ThT signal after 5 h (Fig. 3a and Supplementary Fig. 3a) might be induced by the formation of large aggregates occurring as a result of further aggregate assembly.

With individual AFM images of the aggregates showing comparable morphologies (Fig. 4d–i), we performed a statistical analysis that revealed specific differences in the distributions of thickness and length of fibrils formed in presence and absence of DesBP. Fibrils formed at different time points (0 h, 4 h and 24 h) were used for this analysis. The mica substrates were treated with (3-aminopropyl)triethoxysilane (APTES) to increase the interaction of negatively charged Aβ42 for accurate analysis. In the presence of DesBP, Aβ42 fibrils at 24 h showed higher mean height values (6.3 ± 1.9 nm) than at other times during the aggregation reaction (5.5 ± 1.5, 5.4 ± 1.4, and 5.2 ± 2.1 nm for fibrils at 4 h and 24 h formed in absence of DesBP and at 4 h formed in presence of DesBP, respectively) (Fig. 4k). Further, Aβ42 fibrils formed in presence of DesBP showed wider distributions in the height values (Fig. 4j), implying a greater extent of polymorphism of the fibrils themselves. The mean length was calculated using the log-normal distribution is defined by Eq. 1 (see “Materials and methods” section), as many size measurements in nature tend to have a log-normal distribution, for instance, the lengths of inert appendages (hair, nails, and teeth) in biology, or the lengths of amyloid fibrils^66–69^. This analysis showed significant differences in the mean length of fibrils at 4 h and 24 h formed in absence of DesBP (122 ± 2 and 297 ± 10 nm, respectively), while there was no difference in fibrils at 4 h and 24 h formed in presence of DesBP (148  ±  4 and 176  ±  4 nm, respectively).

As the concentration of DesBP was increased at Aβ42:DesBP ratios above 1:1, the intensity of the ThT fluorescence was suppressed (Supplementary Fig. 3a) and the CD spectra showed structural changes towards lower β-sheet content (Supplementary Fig. 9a, b). The morphologies of these aggregates were analysed by AFM measurements (Supplementary Fig. 9c–g). Aβ42 aggregates were prepared at 10 μM with DesBP at concentration of 0, 20, 160 μM after 24 h incubation at 37 °C without APTES treatment. These images showed morphological changes from fibrilar to non-fibrillar aggregates as the DesBP concentration was increased. We also confirmed that DesBP alone at high concentrations did not form aggregates after 24 h incubation.

To compare the stability of aggregates formed in the presence of 20 μM DesBP, the solution was incubated on ice overnight after CD measurements at 37 °C (Supplementary Fig. 10). At low temperatures, the CD signal at approximately 218 nm immediately decreased, as often occurrs in disordered protein or peptide monomers^70–72^, which is mainly caused by temperature dependent intramolecular hydrophobic interactions. Further incubation on ice showed reversible changes of the CD spectra to more disordered states, as observed for the cold denaturation of aggregates^72,73^, suggesting that these aggregates may have less stability and easily dissociate to monomer state.

In order to find out whether or not DesBP was co-aggregated with Aβ42, mass spectrometry was performed (Fig. 4n). Aggregates formed in the absence and presence of 20 μM DesBP after 24 h incubation (sample 2 and 4 respectively) were collected using ultra-centrifugation and carefully washed with buffer to remove peptide monomers in solution (see “Materials and methods” section). The pellet was then dissociated in 8 M Gdn-HCl, and MALDI-TOF-MS was performed to check whether DesBP was forming aggregates. Considering the absorption of DesBP on the surface of Aβ42 fibrils, one extra sample to which DesBP was added after preparation of Aβ42 fibrils and incubated for 3 h (sample 3) was prepared as control. MALDI-TOF MS analysis showed a small DesBP peak in sample 3, indicating that DesBP can bind the fibrils. However, sample 4 showed a much larger DesBP peak, confirming that most DesBP is incorporated into the Aβ42 aggregates when DesBP is present from the beginning of the aggregation reaction.

Taken together, these results show that DesBP is incorporated in the aggregated species during the aggregation process, producing shorter, thicker and less stable fibrils.

### Characterisation of the effects of DesBP on the morphology of Zn^2+^-stabilised Aβ40 oligomers

In order to obtain insight into the effects of DesBP on the physico-chemical properties of the soluble oligomeric species formed during the aggregation process of Aβ, we performed 8-anilino-1-naphthalenesulfonic acid (ANS) fluorescence and dynamic light scattering (DLS) assays on a recently characterized model system consisting of Zn^2+^-stabilised Aβ40 oligomers^74^, which was chosen because it is very challenging to obtain well-characterised stable Aβ42 oligomers. Both assays showed significant changes in the ANS fluorescence and in the size of the oligomers in the presence of DesBP (Fig. 5), indicating that DesBP affects the morphology of these oligomers. At high Aβ40:DesBP ratios (1:0–1:2), the ANS fluorescence is consistent with the maximum ThT intensity observed for Aβ42 fibrils (Fig. 3c).

Since the ANS intensity does not change linearly with the DesBP concentration, it is possible that both changes in size induced by clustering the oligomers, and structural changes that alter the states of exposed surfaces could have occurred. At low Aβ40:DesBP ratios (1:4–1:32), the ANS fluorescence showed large differences with the ThT intensity of fully formed fibrils, and DLS and turbidimetry measurements indicate the formation of larger aggregates (Fig. 5 grey bars, and Supplementary Fig. 11a–c, respectively). We also confirmed that the aggregates are not fibrillar using a ThT assay (Supplementary Fig. 11d). As the concentration of DesBP was increased, oligomers tend to cluster in a disordered manner.

Taken together, these results indicate that DesBP interacts with Zn^2+^-stabilised Aβ40 oligomers, a finding that, considering also the initial speed up of Aβ42 aggregation (Fig. 3a, b), suggests that it may interact with soluble oligomeric species populated during the Aβ42 aggregation process.

### Effects of DesBP on Aβ42-mediated dysfunction in a *C. elegans* model of Alzheimer’s disease

We further evaluated the effects of DesBP on the formation of toxic Aβ42 species in a *C. elegans* model of Aβ42-mediated dysfunction, denoted GMC101 (termed the ‘Aβ worm model’)^75^. In this model, overexpression of Aβ42 leads to an age-dependent formation of inclusions and to muscle paralysis. We showed previously the utility of this model in drug discovery for Alzheimer’s disease by using either small molecules^59,60^ or designed antibodies^32^. In order to investigate the effect of DesBP in vivo in this worm model, we tested it using a recently developed protocol that allows to deliver protein molecules, including antibodies, to specific tissues in the animals, by encapsulating them into lipid vesicles^76^. We used this protocol in combination with the WF-NTP screening method, which allows multi-parametric and fully automated behavioral analysis of *C. elegans* fitness^77,78^.

We thus administered DesBP to Aβ worms at day 4 (D4) of adulthood, when Aβ42 aggregates are already formed, and paralysis is ongoing, and compared the resulting effects of the addition of DesBP to those observed in a control worm model, N2 (see “Materials and methods” section). By administering DesBP in this manner, we could observe a dose-dependent protective effect of the bicyclic peptide that was maximum at 40 μM (Fig. 6). On the other hand, the effects of the same concentration of DesBP to control N2 worms were negligible when compared to the effect observed on Aβ worms (Supplementary Fig. 12).

## Discussion

It has been recently suggested that a particularly effective way of modulating the aggregation process of Aβ42 is to find compounds, such as antibodies, molecular chaperones and small molecules, that bind with low affinity the monomeric forms, and with high affinity aggregated forms, as such compounds can work at low stoichiometries^34,44,79,80^. Following this strategy, in this work we have reported the design of a bicyclic peptide, called DesBP, with a binding affinity for the monomeric form of Aβ42 in the high μM range (Fig. 2c), but that in in vitro aggregation experiments at low molar equivalents of DesBP (at an Aβ42:DesBP ratio of 4:1) was able to re-direct the Aβ42 aggregation process towards the formation of modified aggregated species.

We have found that at these low concentrations of DesBP, the Aβ42 aggregates at the end of the aggregation reaction are fibrillar, as shown by CD and AFM analysis (Fig. 4a–i). Mass spectrometry indicated that DesBP was incorporated in such aggregates (Fig. 4n), and a statistical AFM analysis revealed that the fibrils are thicker (Fig. 4j, k) and shorter (Fig. 4l, m) than those formed in the absence of DesBP. At higher DesBP concentrations, the morphology of Aβ42 assemblies are changed to non-fibrillar aggregates (Supplementary Fig. 9) and the concentration of soluble Aβ42 was increased at Aβ42:DesBP ratios below 1:2 (Supplementary Fig. 8), which resulted in changes detectable in the CD spectra. In addition, aggregates formed in the presence of 20 μM DesBP are readily dissociated at low temperatures. The formation of optimal configurations, such as backbone hydrogen bonding networks^81^ and steric zipper side chain packing^82^, that contribute to achieve high stability of amyloid fibrils^83^ appears thus to be disturbed by DesBP, decreasing the stability of the assemblies.

These results support the conclusion that increasing concentrations of DesBP can alter the morphology of Aβ42 aggregates towards increasing disorder (Fig. 7), which are of reduced cytotoxicity (Fig. 6). In the presence of 0.25 molar equivalents of DesBP, the reduction of average length of fibrils formed at 24 h is nearly 60%, consistent with the conclusion that DesBP accelerates Aβ42 aggregation (Fig. 3) by changing the morphologies of the aggregate species (Fig. 4).

In addition, the effects of DesBP on Zn^2+^-stabilised Aβ40 oligomers, which we used as models of the variety of soluble oligomer species that may be present in the diseased brain, were analysed using ANS and DLS assays (Fig. 5). These assays showed that both the size and the surface properties of these oligomers are dramatically affected by DesBP. These results suggest that the presence of DesBP may affect the conversion step by which early disordered aggregates reorganise their structures to more stable ordered β-sheet structure^40,84,85^.

The design strategy that we have used to identify DesBP suggests a mechanism of inhibition of Aβ42 aggregation by which this compound binds the C-terminus of Aβ42 (Fig. 1), thus interfering with the Aβ42 aggregation process by driving it towards alternative pathways (Fig. 7). The observation that DesBP also binds Zn^2+^-stabilized Aβ40 oligomers suggests that this bicyclic peptide could also interfere with the aggregation processes of Aβ40 and mixed Aβ40/Aβ42^86^, as well as of truncated forms of Aβ^87^, which are likely to play a role in Alzheimer’s disease.

## Conclusions

We have described a procedure for the rational design of bicyclic peptides and illustrated it by generating one peptide of this type, called DesBP, to bind Aβ42 as a strategy to modulate Aβ42 aggregation. In the design procedure we have also optimized the solubility of DesBP in order to generate a compound that could remain soluble even at high concentrations and that could be readily produced and used in a variety of assays.

We have then shown that DesBP interferes with the Aβ42 aggregation process at low stoichiometries as it does not have high affinity for Aβ42 monomers, but it is capable of affecting early species in the Aβ42 aggregation process and is incorporated in the late aggregated species. The presence of DesBP in Aβ42 aggregates appears to disturb the well-ordered β-sheet structure of Aβ42 fibrils, and change their morphologies to more disordered aggregates. The redirection of the aggregation process of Aβ42 by DesBP was consistent with in vivo experiments in a *C. elegans* model of Alzheimer’s disease, which showed that DesBP suppresses the toxicity associated with Aβ42-aggregation in this animal model.

Taken together, these results illustrate how the rational design procedure that we have described enables bicyclic peptides to be generated that are able to interfere with the Aβ42 aggregation process and reduce its associated toxicity.

## Materials and methods

### Reagents

Thioflavin T (ThT) UltraPure Grade (≥ 95%) was purchased from Eurogentec Ltd. All the other reagents, including Tris-(bromomethyl)benzene (TBMB), were purchased from Sigma Aldrich.

### Rational design of the bicyclic peptide

The bicyclic peptide sequence can be regarded as divided into four regions, separated by the three cysteine residues required for bicyclisation. The two central regions were designed to bind the target epitope (Fig. 1b), while the amino acid sequence of the N- and C-terminal regions retained some motifs that were found to facilitate the bicyclisation reaction (i.e. AA at the N-terminus and GG at the C-terminus), and were further decorated with charged residues to enhance solubility. The length of each central region (i.e. the binding sites) was limited to six residues, as attempts to carry out the bicyclisation reaction with longer sequences, or without the AA and GG motifs at the termini were unsuccessful (data not shown).

The sequences of the two central regions were designed with the cascade method^31^ to bind respectively to Aβ42 at residues 31–36 and 38–42, which are consecutive epitopes along the Aβ42 sequence (position 37 corresponds to the cysteine residue in the designed peptide, Fig. 1b). We reasoned that this epitope choice should provide the DesBP with more chances to engage with the C-terminus of Aβ42, albeit the cyclic nature of the peptide makes it unlikely that all residues in the designed sequence will simultaneously bind to the Aβ monomer, as such binding would require the latter to curl around the cyclic structure of the peptide (Fig. 1c). The identity of the charged residues at the termini were determined by maximizing the CamSol intrinsic solubility score^56^, and unsurprisingly the sign of the charges matches that of the designed binding region.

### Recombinant expression of Aβ42

The recombinant Aβ42 peptide (MDAEFRHDSGY EVHHQKLVFF AEDVGSNKGA IIGLMVGGVV IA) was expressed in the *Escherichia coli* BL21 Gold (DE3) strain (Stratagene) and purified as described previously^88^. Briefly, the purification procedure involved sonication of *E. coli* cells, dissolution of inclusion bodies in 8 M urea, ion exchange in batch mode on diethylaminoethyl cellulose resin, and lyophilization. The lyophilized fractions were further purified using a Superdex 75 h 26/60 column (GE Healthcare), and eluates were analyzed using SDS-polyacrylamide gel electrophoresis for the presence of the desired product. The fractions containing the recombinant protein were combined, frozen using liquid nitrogen, and lyophilized again.

### Peptide synthesis

Linear peptides were purchased from ChinaPeptides^89^. For cyclization, peptides were dissolved in reaction buffer (20 mM NH_4_HCO_3_, 5 mM EDTA, pH 8.0) at 625 μM. One quarter volume of 5 mM TBMB in 100% acetonitrile was added to obtain a final concentration of 500 μM peptide and 1 mM TBMB and incubated for 1 h at 30 °C. The cyclised peptide was purified by reversed-phase chromatography on a C18 column using H_2_O/0.08% trifluoroacetic acid (TFA) and acetonitrile/0.08% TFA as solvents. The column used was a GRACE VYDAC C18 (218TP) column 22 × 250 mm. The correct mass was confirmed by analytical LC/MS (Xevo).

### Fluorescence assay

Monomeric Aβ42 peptide solutions were prepared by dissolving the lyophilized peptide in 6 M GuHCl. A Superdex 75 10/300 GL column (GE Healthcare) was used to purify Aβ42 monomers from Aβ42 oligomers and salt with a flow rate of 0.5 ml/min, and eluted in 20 mM sodium phosphate buffer (pH 8) with 200 μM EDTA. By collecting the centres of the peaks, the monomeric Aβ42 concentrations were determined from the integrated peak area with ε_280_ = 1,495 L mol^−1^ cm^−1^. Aβ42 monomers were then diluted to the target concentration with buffer and 20 μM ThT was added from a 1 mM stock. All samples were prepared in low-binding Eppendorf tubes on ice using pipetting to avoid the formation of air bubbles. Sample were pipetted into a 96-well half-area at 80 μl per well, using low-binding polyethylene glycol coating plates (Corning 3881). Assays were carried out at 37 °C under quiescent conditions in a plate reader (Fluostar Optima; BMG Labtech). ThT fluorescence was measured through the bottom of the plate with 440 nm excitation and 480 nm emission filters, with five repeats per sample. In the seeding experiments we used 10% preformed fibrils.

### Kinetic analysis

The normalised ThT curves were fitted using the fitting platform AmyloFit^90^, which is freely accessible online (https://www.amylofit.ch.cam.ac.uk/). Because the morphology of the aggregates is affected by DesBP and the ThT intensity is thus changed, a quantitative analysis was applied only for Aβ42 aggregation in the presence of small amounts of DesBP.

### Oligomer assay

Lyophilized Aβ40 at 0.5 mg/ml was solubilized overnight in 300 µl HFIP to obtain the monomeric form. The solvent was then evaporated under a gentle flow of nitrogen gas. The peptide was resuspended in DMSO at a concentration of 2.2 mM and sonicated twice for 10 min at room temperature. The Aβ40 peptide was then dilute in 20 mM sodium phosphate buffer, at pH 6.9, with 200 µM ZnCl_2_ to a final concentration of Aβ40 of 100 µM, incubated at 20 °C for 20 h and centrifuged at 15,000 g for 15 min at 20 °C. The pellet containing the oligomers was resuspended in phosphate buffer. DesBP was centrifuged at 20 °C for 1 h at 435,000 g and then incubated in isolation or in combination with the Aβ40 oligomers at different ratios for 2 h at room temperature.

### ANS binding

Samples containing Aβ40 oligomers incubated in isolation or combination with DesBP were subjected to ANS binding measurements. ANS spectra were recorded using a plate reader (BMG Labtech, Aylesbury, UK) with excitation at 380 nm. The measurement was acquired at 25 °C in phosphate buffer. The final concentration of oligomers was 5 µM with a threefold excess of ANS. Samples were measured in duplicate and five independent experiments were performed.

### Dynamic and static light scattering

Samples containing Aβ40 oligomers were incubated in isolation or combination with DesBP and subjected to DLS and SLS measurements. The light scattering measurements were performed on a Zetasizer Nano S instrument (Malvern Instruments, Malvern, UK) working in backscattering mode at 173°, equipped with a light source with a wavelength of 633 nm and a Peltier temperature controller at 25 °C. DesBP was centrifuged at 20 °C for 1 h at 435,000 g and incubated with the Aβ40 oligomers for 1 h at room temperature at different ratios.

### CD spectroscopy

Far-UV CD spectra of proteins and peptides in soluble and insoluble states were measured with a J-820 spectropolarimeter (Jasco, Japan) using a cell with a light path of 1 mm at each condition. Individual Aβ42 solutions were prepared at 10 μM for CD measurements. The CD signals between 195 and 250 nm were expressed as mean residue ellipticity [*θ*] (deg cm^2^ dmol^−1^). Temperature regulation was carried out using a PFD-425S Peltier-unit (Jasco, Japan).

### Atomic force microscopy

Atomic force microscopy (AFM) measurements were performed in air on positively functionalized mica surface, which was incubated for 1 min after cleaving with a 10 μl drop of 0.05% (v/v) (3-Aminopropyl)triethoxysilane (APTES) (Fluka) in Milli-Q water at room temperature, rinsed with Milli-Q water and dried with a flow of nitrogen. AFM samples were prepared at room temperature by deposition of a 10 μl aliquot of 10 μM solution for 5 min, followed by rinsing ultrapure water and dried by a flow of nitrogen.

Imaging was performed in intermittent contact mode on a JPK Nanowizard II microscope in ambient conditions, with integral gain 120 Hz, post-gain 0.008 Hz, 0.3 Hz line-rate for 4 × 4 μm images. The images flattening and statistical analysis were carried out using SPIP (Image metrology). The fibril cross sections were traced manually in order to determine the length and height of the fibrils. The cross section of each fibril was described by tracing manually along the ridges, and the length and height of fibrils were elucidated. In the statistical analysis of the results, we used a log-normal distribution

1 $$f(L)=\frac{A}{L\sigma\sqrt 2 }e^{-[{{\text{ln}(L)-\mu}]^{2}/2\sigma^{2} }}$$

where *L* is the normalised length of the fibril, *μ* and *σ* are the mean and the standard deviation of the natural logarithm of *L*, and *A* is a constant of normalisation.

### Quantification of residual Aβ42 monomers

200 μL of Aβ42 solution at a given time point was centrifuged at 20 °C for 30 min at 100,000 rpm (435,000 g) and 100 μL of supernatant was separated. The mass of Aβ42 in supernatant was quantified by high performance liquid chromatography (HPLC) using Aeris Widepore XB-C18 column (3.6 μm, 250 mm × 4.6 mm, Phenomenex) connected to an Agilent 1260 Infinity system in acetonitrile containing 0.2% ammonia at 50 °C and a flow rate of 0.2 ml/min. Absorbance was measured at 280 nm. To remove the deprotonated DesBP, the column was also washed with acetonitrile containing 0.1% TFA.

### Mass spectrometry

Aβ42 aggregates were prepared by 1 day incubation at 37 °C in the presence and absence of DesBP. To remove soluble Aβ42 monomers, the samples were centrifuged at 20 °C for 1 h at 100,000 rpm (435,000 g) and the pellets were washed with 0.1% SDS. The samples were further spun down and resuspended in buffer repeatedly to remove SDS. After repeating the washing three time, pellets were dissolved in 8 M Gdn-HCl solution at pH 9.0 for monomerization (3 days at room temperature). Mass spectrometry was performed using MALDI-TOF MS (Bruker ultrafleXtreme). The samples were desalted before MS using C18 Zip Tip; 4 × 2 μl up-down, wash 10 ×, elute with 1.5 μl matrix in 50% MeCN/0.1% TFA. At a sample of the aggregates formed in the presence of DesBP, sampling size was increased by 1.5 times because of its too weak signal.

### NMR measurements

For NMR analyses, uniformly labeled ^15^N-Aβ42 was purchased from rPeptide. Lyophilized powder of ^15^N-Aβ42 was dissolved at an approximate concentration of 2 mM NaOH solution and then collected and stored in aliquots at − 80 °C until use. ^15^N-Aβ42 was diluted to 15 μM of with 20 mM sodium phosphate buffer (pH 7.4), 10% (v/v) D_2_O, and 1 mM DesBP. The pH of the mixture was checked immediately before measurement. 32 scans were taken for each spectrum using a 500 MHz NMR AVANCE-500 spectrometer equipped with a cryogenic probe (Bruker) at 5 °C to ensure that the Aβ42 peptide remained monomeric during data acquisition. Residue assignments were taken from previously published work^91^. Chemical shift perturbations (CSP) were calculated as Δδ = ((Δδ_N_/6.4)^2^ + (Δδ_HN_)^2^)^1/2^^92^. NMR spectra were processed by TopSpin 2.1 (Bruker). Resonance assignment and intensity calculations were performed using the Sparky Program.

### Isothermal titration calorimetry (ITC)

ITC measurements for binding between Aβ42 and DesBP were performed with an ITC-200 instrument (Malvern). The DesBP at 1 mM with 20 mM sodium phosphate buffer (pH 8.0) and 200 μM EDTA was injected into the sample cell containing approximately 200 μl of 10 μM Aβ42. ITC titrations were performed at 15 °C by using 2 μl injection with a total of 19 injections with stirring at 350 rpm. The low temperature, stirring speed, and concentration of Aβ42 and short initial delay (120 s) and spacing time (120 s) were selected to avoid the aggregation of Aβ42. The observed thermogram did not show any large baseline changes caused by aggregation reaction of peptides and the structure of peptide after ITC measurements was confirmed by CD spectrometry. Data were fitted with a one-binding site model using Microcal Origin software.

### Bio-layer interferometry (BLI)

To test for direct peptide-peptide interaction, biolayer interferometry (BLI) was performed on the Octet RED96 System (ForteBio, Menlo Park, USA). DesBP was used as interaction partner for sensor-coupled Aβ42. Coupled sensors were first dipped into ForteBio kinetics buffer (PBS, 0.1% BSA, 0.02% Tween20 and 0.05% sodium azide, pH 7.4) to establish a stable baseline, then into different concentrations of DesBP and finally in pure kinetics buffer again to monitor dissociation. Both association and dissociation processes were monitored at 30 °C over 50 and 200 s, respectively. In the data analysis, all curves were globally fit with a 1:1 binding model.

### *C. elegans* experiments

*Media* Standard conditions were used for the propagation of *C. elegans*^93^. Briefly, the animals were synchronized by hypochlorite bleaching, hatched overnight in M9 (3 g/l KH_2_PO_4_, 6 g/l Na_2_HPO_4_, 5 g/l NaCl, 1 µM MgSO_4_) buffer, and subsequently cultured at 20 °C on nematode growth medium (NGM) (CaCl_2_ 1 mM, MgSO_4_ 1 mM, cholesterol 5 µg/ml, 250 µM KH_2_PO_4_ pH 6, Agar 17 g/L, NaCl 3 g/l, casein 7.5 g/l) plates seeded with the *E. coli* strain OP50. Saturated cultures of OP50 were grown by inoculating 50 mL of LB medium (tryptone 10 g/l, NaCl 10 g/l, yeast extract 5 g/l) with OP50 and incubating the culture for 16 h at 37 °C. NGM plates were seeded with bacteria by adding 350 µl of saturated OP50 to each plate and leaving the plates at 20 °C for 2–3 days. On day 3 after synchronization, the animals were placed on NGM plates containing 5-fluoro-2′deoxy-uridine (FUDR) (75 μM, unless stated otherwise) to inhibit the growth of offspring. FUDR plates were seeded with bacteria by adding 350 µl of 10 × concentrated OP50 solution to ensure starvation did not occur for the lifespan of the worm. Concentrated OP50 solution was obtained by centrifuging 1 L of saturated OP50 culture at 5,000 rpm for 15 min and suspending the resultant pellet in 100 ml sterile water.

*Tracking analysis* Analysis was carried out as described previously^77,78^. Briefly, we used custom software written in Python (Python Software Foundation) called the Wide Field-of-view Nematode Tracking Platform (WF-NTP)^77,78^. Our code initially detects and subtracts the background, consisting of non-moving objects such as small particles and shadows from the agar plate. After this operation, the remaining labeled regions are identified as individual worms and the positions of such regions are then stored for each frame. The eccentricity of each tracked worm, a measure of the ratio of the major and minor ellipse axes, can then be used to estimate worm body bending as a function of time. Through this method, individual worms can be tracked over time, and plots of their movement can be extracted to give visual information about their mobility levels.

*Strains of C. elegans *The following strains of *C. elegans* were used: dvIs100 [unc-54p::A-beta-1-42::unc-54 3′-UTR + mtl-2p::GFP] (GMC101), which produces constitutive expression of GFP in intestinal cells; unc-54p::A-beta-1-42 which expresses full-length human Aβ42 peptide in body-wall muscle cells that aggregates in vivo; shifting L4 or young adult animals from 20 to 25 °C causes paralysis^75^. The *C. elegans* N2 strain was used as control. Generation time is about 3 days. Isolated from mushroom compost near Bristol, England^93^.

*Automated motility assay on agar plates* All *C. elegans* populations were cultured at 20 °C and developmentally synchronized by hypochlorite bleaching. Unsynchronized animals were washed off NGM plates using M9 buffer and centrifuged at 2,000 rpm for 2 min, the supernatant was removed to leave 2 ml of M9. 1 ml of hypochlorite bleaching solution was then added and the mixture agitated for 210 s before being diluted to 15 ml using M9, animals were then subjected to 5 rounds of centrifugation at 2,000 rpm for 2 min followed by removing the supernatant and suspending in 15 ml of M9 to dilute out the hypochlorite bleaching solution. After 5 washing cycles the animals were transferred to 12 well tissue culture plates and allowed to hatch overnight, development was arrested at L1 larval stage due to a lack of food. The now synchronized animals were then transferred to OP50 seeded NGM plates and allowed to develop for 64–72 h before being transferred to seeded FUDR plates. At defined ages, the animals were washed off the plates with M9 buffer and incubated with specific bicyclic peptides for 6 h. Worms were then transferred on FUDR plates and let recover overnight. The morning after, the worms were spread over an OP-50 un-seeded 9 cm plate, after which their movements were recorded at 20 fps using a rationally designed microscopic method, for 2 min^77^. Up to 2,000 animals were screened per condition per time point for each experiment unless stated otherwise. One experiment that is representative of the three measured is shown in the figure. Videos were analysed using a custom made tracking code^77^.

*Transduction protocol* The transduction protocol was carried out as previously described^76^. About 500 *C. elegans* worms were incubated in M9 with 20 μM bicyclic peptide and 40 μl PulsIn (40–70 μM) (PolyPlus tranfection SA, Illkirch-Graffenstaden, France) in a final volume of 1 ml. Motility measurements were carried 24 h after transduction. All experiments were carried out in triplicate. As control we considered wild type worms.

## Supplementary information

Supplementary Information.
