# Supplementary material for: A rationally designed bicyclic peptide remodels Aβ42 aggregation in vitro and reduces its toxicity in a worm model of Alzheimer’s disease
Source: Sci Rep. 2020 Sep 17;10:15280. doi: 10.1038/s41598-020-69626-3 (PMC7498612; doi:10.1038/s41598-020-69626-3)
Supplement: Supplementary file 1 — Supplementary Information. [file 41598_2020_69626_MOESM1_ESM.pdf]

SUPPORTING INFORMATION

A rationally designed bicyclic peptide remodels Aβ42 aggregation *in vitro* and reduces its toxicity in a worm model of Alzheimer's disease

Tatsuya Ikenoue, Francesco A. Aprile, Pietro Sormanni, Francesco S. Ruggeri, Michele Perni, Gabriella T. Heller, Christian P. Haas, Christoph Middel, Ryan Limbocker, Benedetta Mannini, Thomas C. T. Michaels, Tuomas P. J. Knowles, Christopher M. Dobson and Michele Vendruscolo

Centre for Misfolding Diseases, Department of Chemistry,

University of Cambridge, Cambridge CB2 1EW, UK

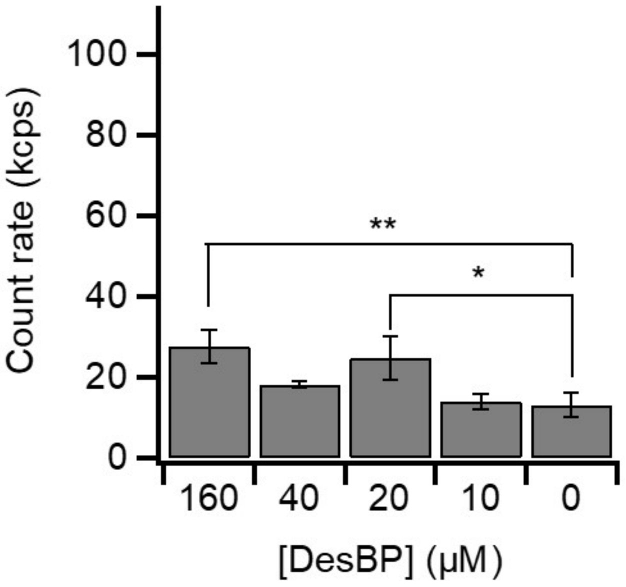

Supplementary Figure 1. Static light scattering measurements to confirm that DesBP remains in its monomeric state in phosphate buffer. Similar count rates of solution in the presence and absence of DesBP indicate that DesBP is monomeric at all the concentrations tested (0 to 160 μM). The symbols * and ** indicate $p < 0.05$ and 0.01, respectively.

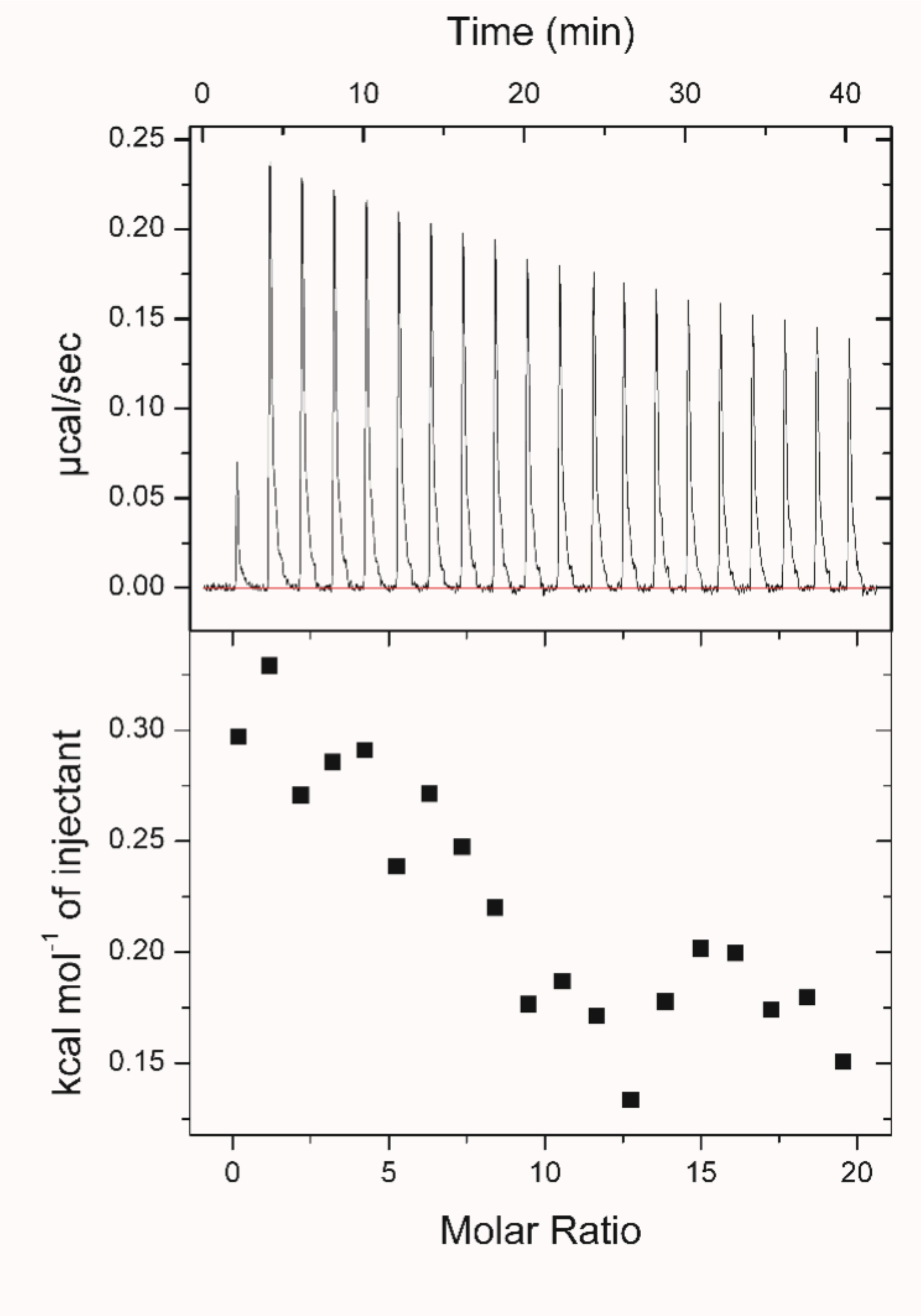

Supplementary Figure 2. ITC thermogram of titration of DesBP to Aβ42 monomers. 1 mM DesBP solution in syringe was titrated to 10 µM of Aβ42 monomer solution in sample cell at 15 °C.

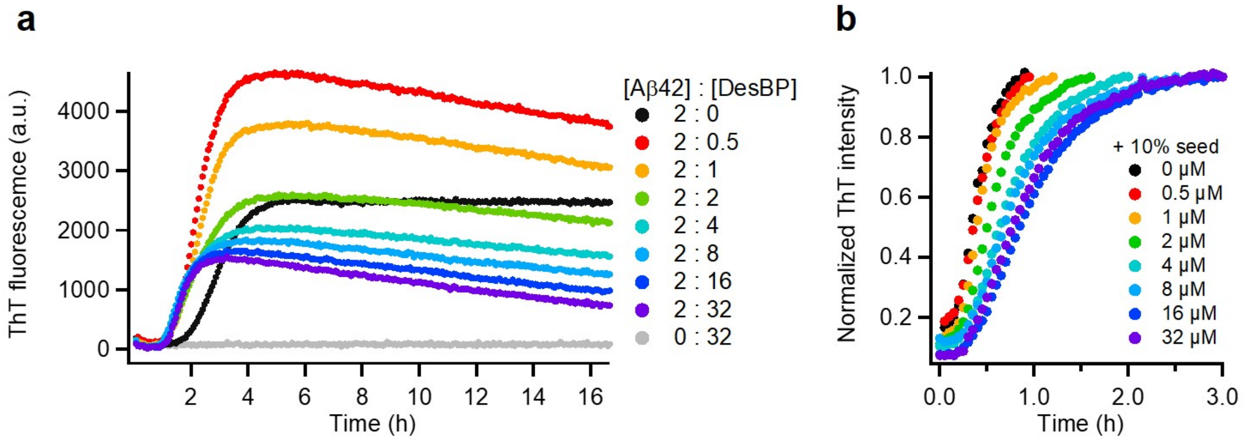

Supplementary Figure 3. ThT profiles for the kinetic analysis. Raw data of ThT fluorescence of spontaneous Aβ42 aggregation used for **Figure 3a** (**a**) and normalised kinetic profile of seeded Aβ42 aggregation (**b**), in the absence or presence of various concentrations of DesBP (represented by different colors).

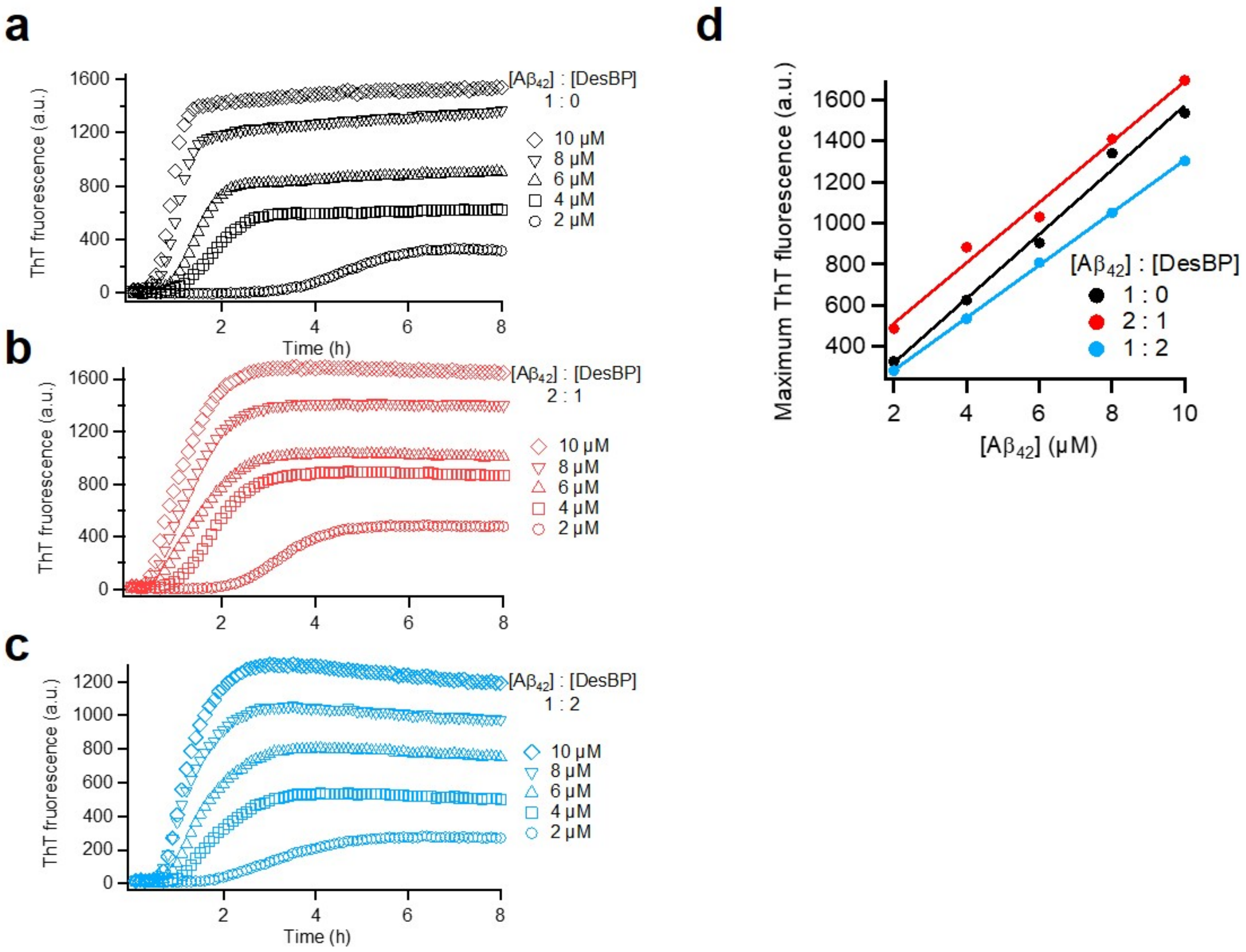

Supplementary Figure 4. Concentration dependence of the inhibition of Aβ42 aggregation by DesBP. (**a-c**) Kinetic profiles of Aβ42 aggregation at various concentrations ranging from 2 to 10 μM at increasing [Aβ42]:[DesBP] ratios: 1:0 (**a**), 2:1 (**b**), and 1:2 (**c**). **(d)** Average maximum ThT fluorescence intensity at increasing [Aβ42]:[DesBP] ratios: 1:0 (black), 2:1 (red), and 1:2 (blue).

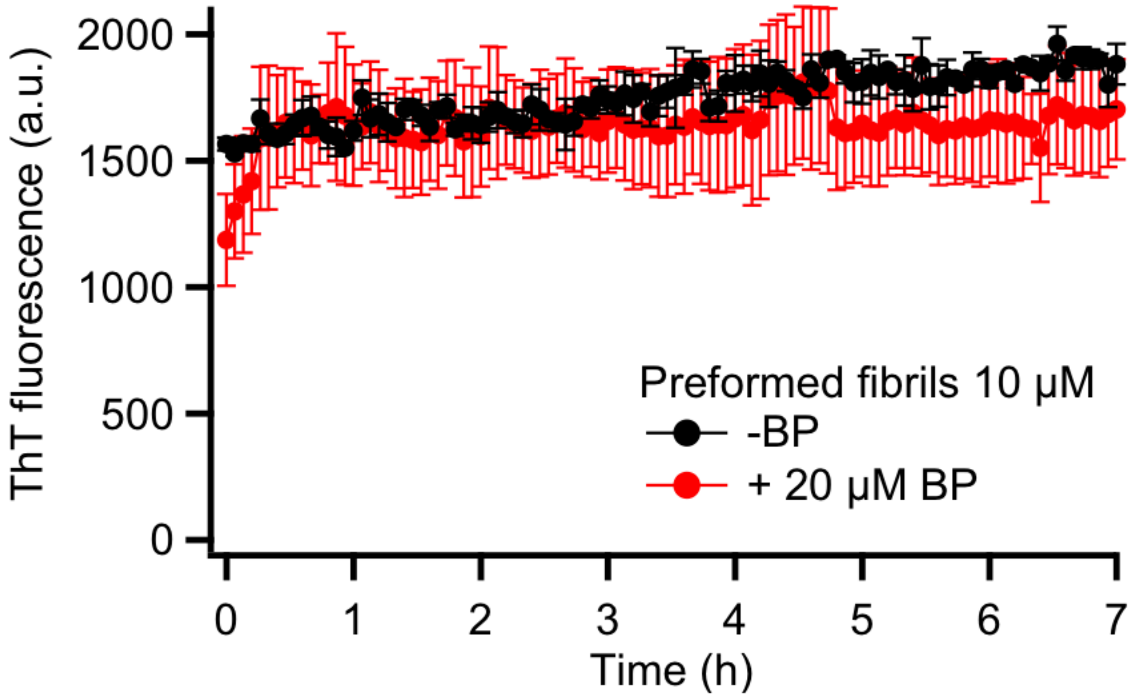

Supplementary Figure 5. ThT assay of preformed Aβ42 fibrils. ThT is added to preformed fibrils with DesBP (red) and without it (black).

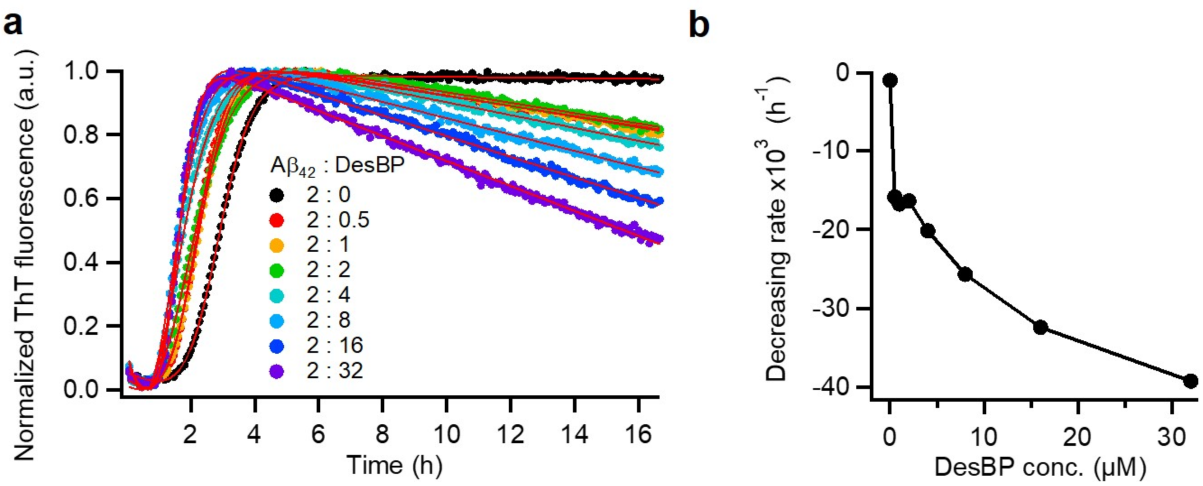

Supplementary Figure 6. Decreasing rate analysis of ThT profiles. (**a**) Normalisation of the ThT profiles shown in **Figure 3a**. Each profile was fitted by equation of ThT signal $S = (M_0 + K(F_0+F_s t))/(1+K)$ where K is the equilibrium constant, M_0 and F_0 are the standard ThT signals of monomeric and fibril state, respectively, and F_s is slope of ThT decreasing rate of formed fibrils. Fitted curves are shown as red solid lines. (**b**) The decreasing rate of ThT signal after reaching maximum intensity estimated from the fitting in panel (**a**).

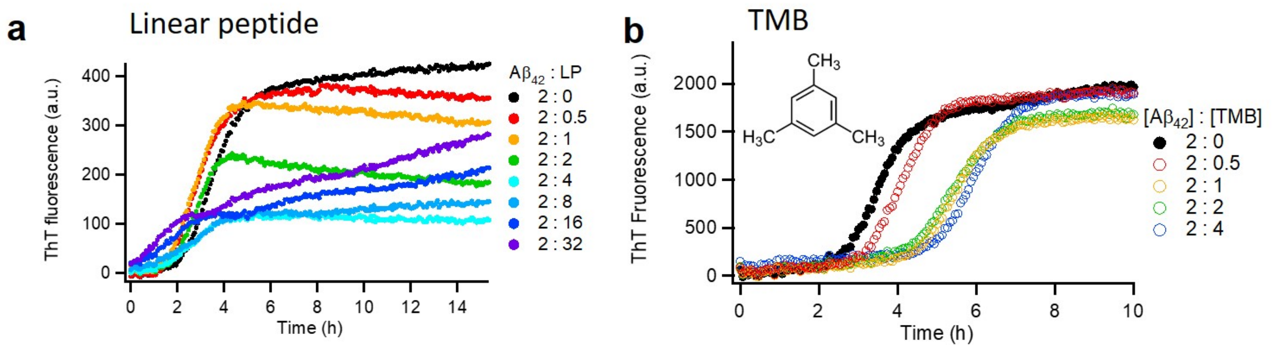

Supplementary Figure 7. ThT assay of Aβ42 in the presence of designed peptide before bicyclisation (linear peptide), and in the present of TMB. Kinetic profiles of Aβ42 aggregation under quiescent conditions at a concentration of 2 μM in the absence or in the presence of various concentrations of the linear designed (**a**) and TMB (**b**) (represented by different colors).

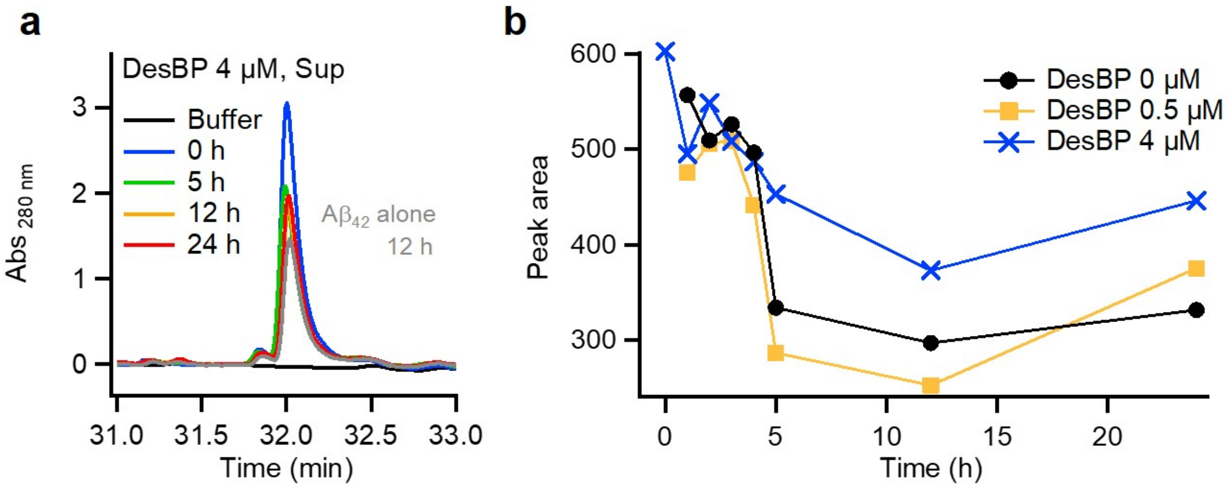

Supplementary Figure 8. HPLC analysis for the quantification of residual Aβ42 monomers. (**a**) Representative chromatogram of the supernatant of 2 μM Aβ42 aggregates formed in the absence and presence of DesBP at various time points. (**b**) Total peak area of each chromatogram.

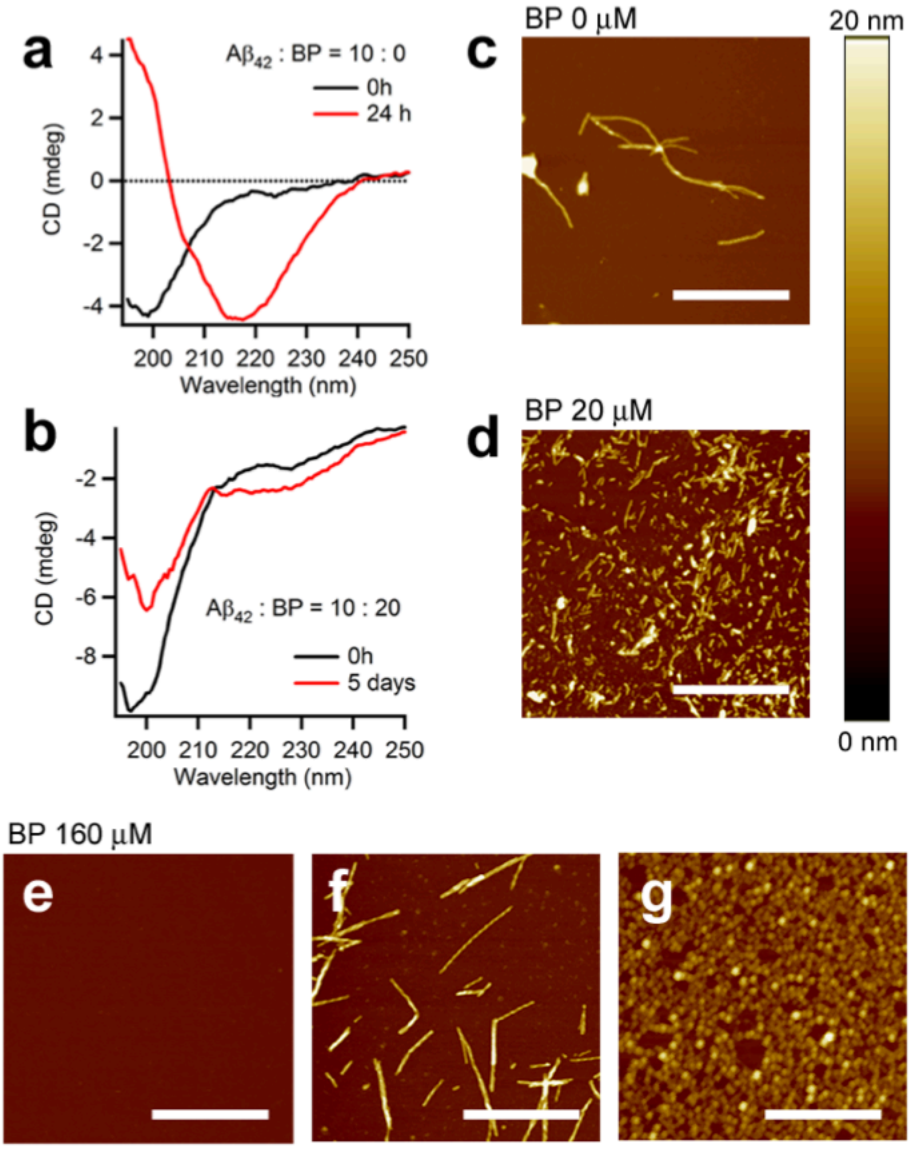

Supplementary Figure 9. Structural features of Aβ42 fibrils formed in the presence of high concentrations of DesBP. (a, b) Far-UV CD spectra of 5 μM Aβ42 aggregates in absence (**a**) and presence (**b**) of 20 μM DesBP. **(c-g)** Representative AFM images for the analysis of the morphology of Aβ42 aggregates at concentration of 0 **(c)**, 20 **(d)**, 160 μM **(g)** of DesBP. Images of 160 μM DesBP alone and fibrils with 160 μM DesBP are shown as control in panels **(e)** and **(f)**, respectively. The scale bar on the AFM images in panels c-g indicates 1 μm, and the bar at the right of panels a-d represents the height.

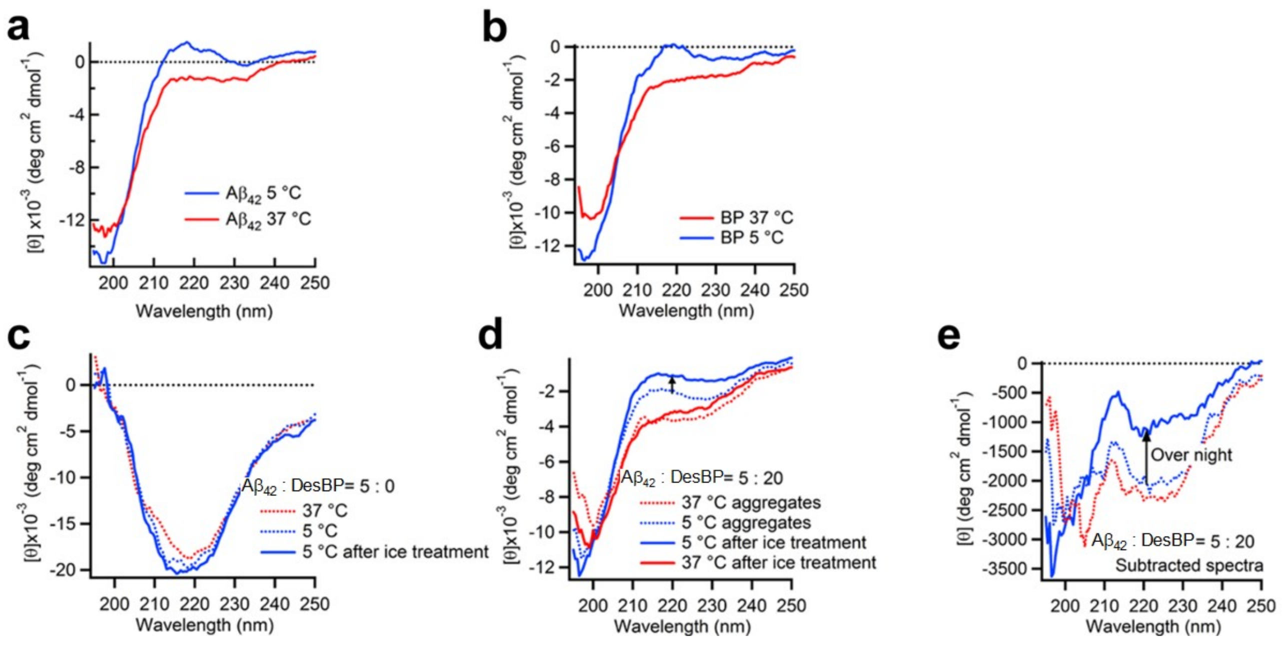

Supplementary Figure 10. Thermal stability of the Aβ42 aggregates in the presence of DesBP. (**a**, **b**) Far-UV CD spectra of Aβ42 monomers in the absence (**a**) and presence of DesBP (**b**) at 5 and 37 ºC. (**c-e**) Cold denaturation examination of Aβ42 aggregates formed in the absence (**c**) and presence (**d**, **e**) of DesBP monitored by far-UV CD. Raw data of mixture of Aβ42 and DesBP is shown in panel (**d**) and spectrum and subtracted Aβ42 spectrum are shown in panel **(e)**.

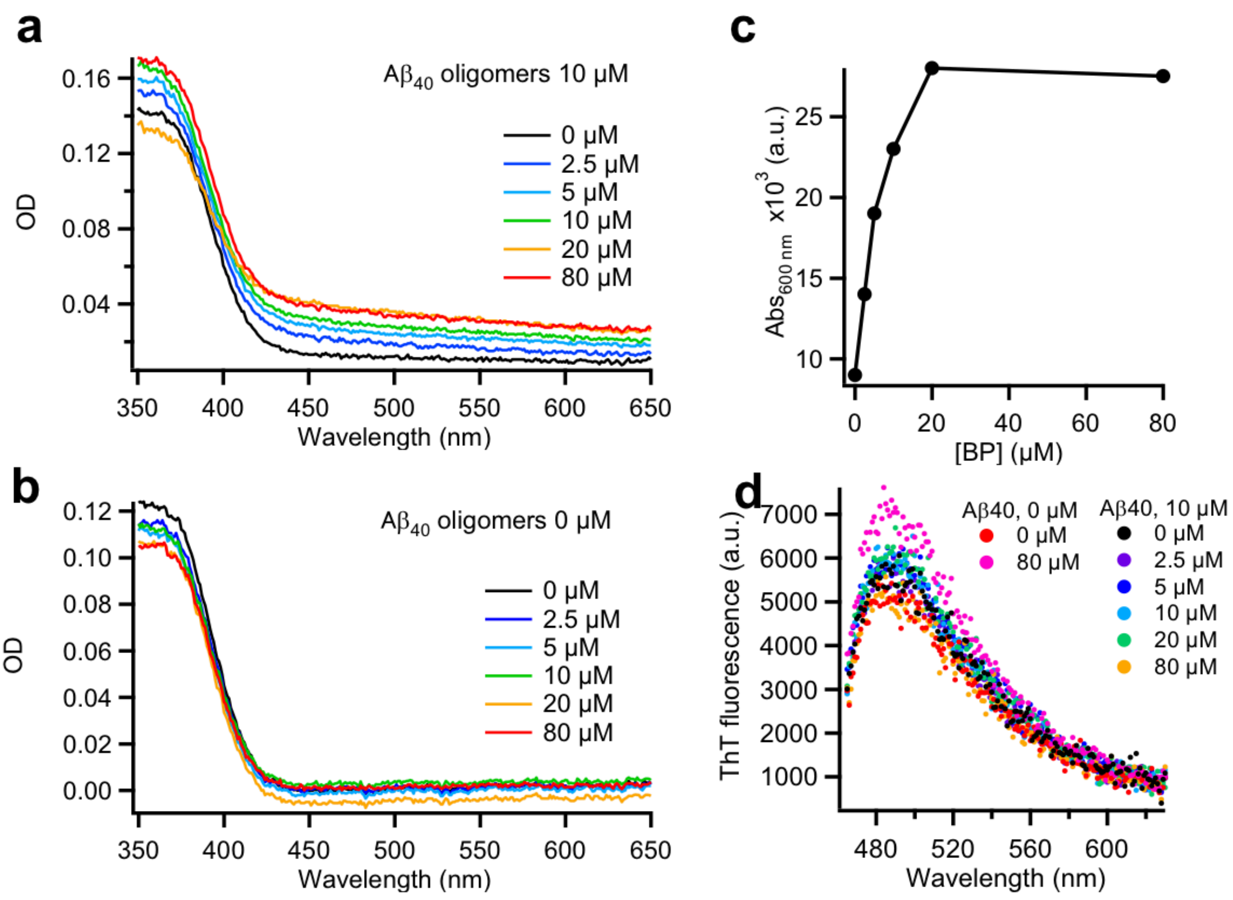

Supplementary Figure 11. Turbidimetry and ThT fluorescence spectra of Aβ_{40} oligomers. (**a**, **b**) Turbidity of 10 µM Aβ_{40} oligomers in the presence of various concentrations of DesBP (**a**) and DesBP alone (**b**) at 25 ºC. Absorption at 600 nm of spectra in panel (**a**) were plotted in panel (**c**). (**d**) ThT fluorescence spectra of Aβ_{40} oligomers in the presence of various concentration of DesBP.

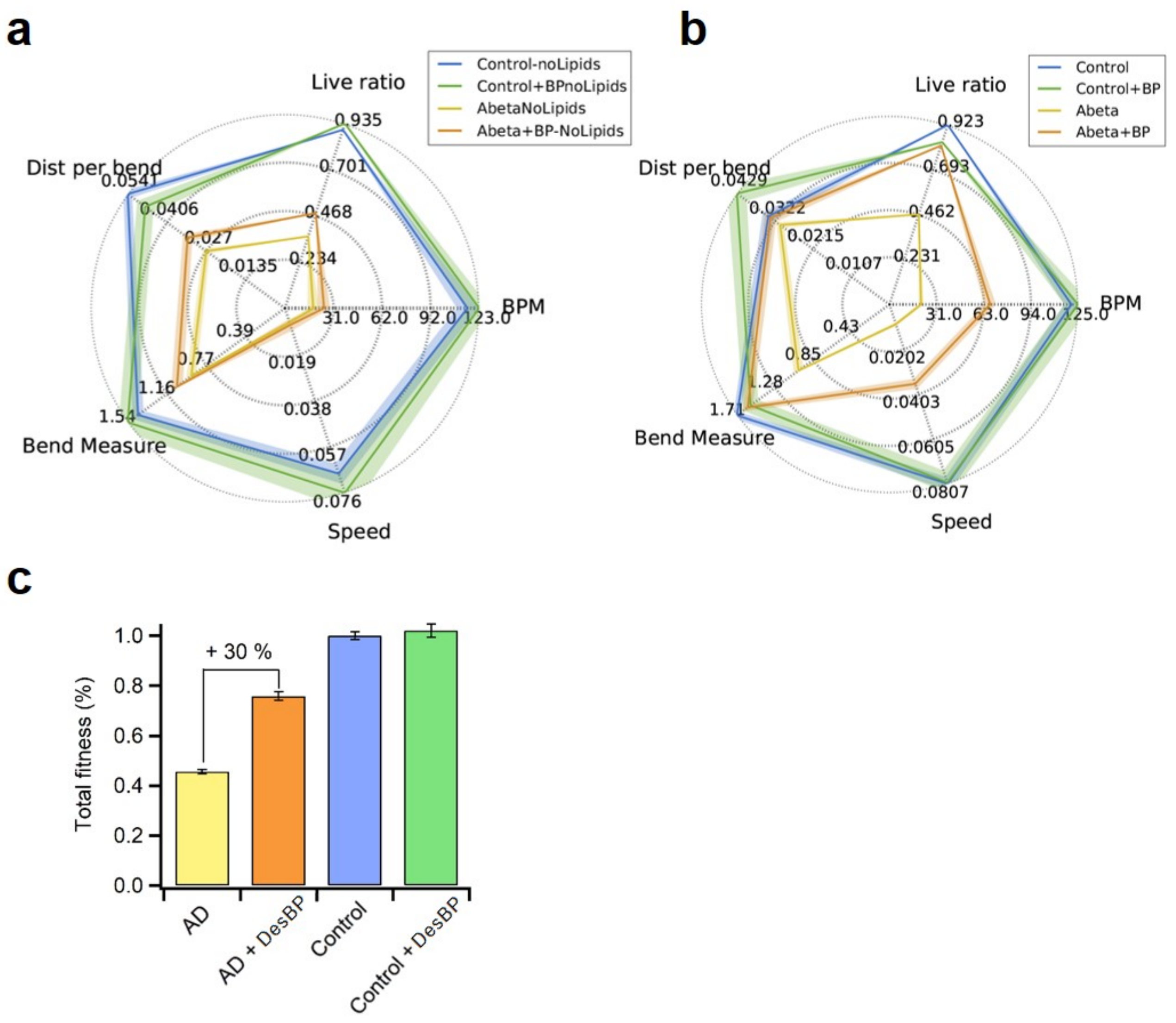

Supplementary Figure 12. Toxicity of DesBP and effects of encapsulation with lipid vesicles. (**a**, **b**) Fingerprint of the measurements of the effect of DesBP in the absence (**a**) and presence (**b**) of lipid vesicle on the motility of native and Aβ42 worm model. (**c**) Total fitness of *C. elegans* in the presence and absence of DesBP with lipid vesicle. N2 worms treated with lipid vesicles (blue) and with DesBP (green) were used as controls.
